# Supplementary material for: Asynchronicity of endemic and emerging mosquito-borne disease outbreaks in the Dominican Republic
Source: Nat Commun. 2021 Jan 8;12:151. doi: 10.1038/s41467-020-20391-x (PMC7794562; doi:10.1038/s41467-020-20391-x)
Supplement: Supplementary file 1 — Supplementary Information [file 41467_2020_20391_MOESM1_ESM.pdf]

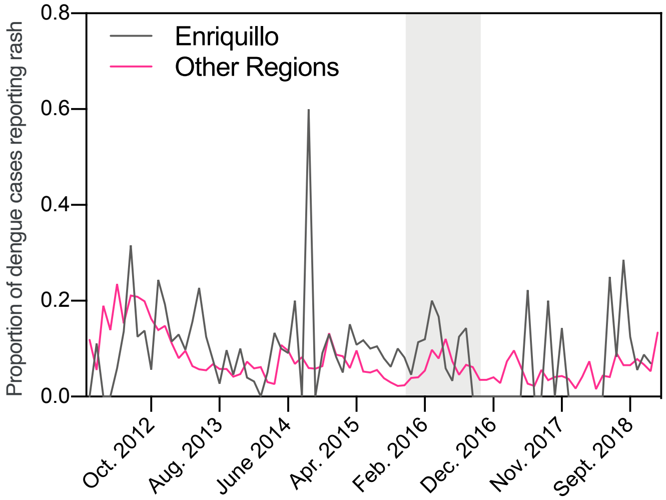


**Supplementary Figure 1: The proportion of dengue cases who reported experiencing a rash peaked earlier in the south-western region Enriquillo compared to the rest of the country.** The number of dengue cases with rash were summed for each month. The gray box denotes when the Zika outbreak occurred.

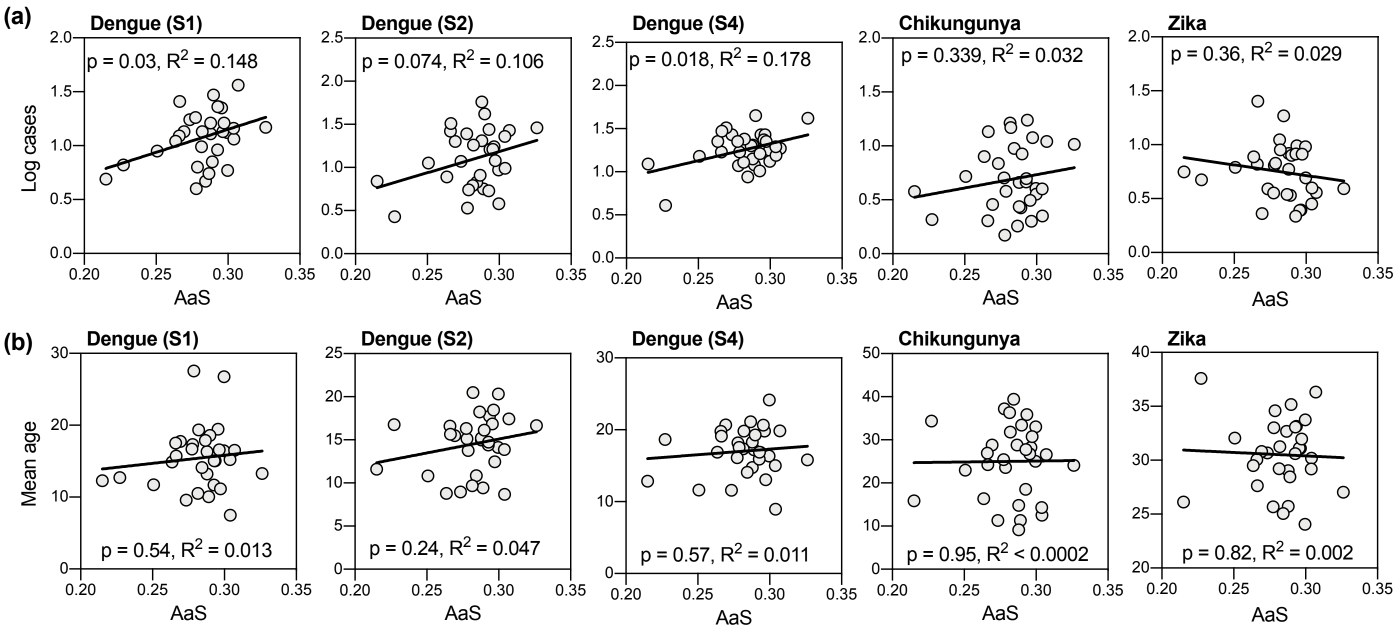


**Supplementary Figure 2: Minimal correlation between adjusted log attack rates, force of infection, and the average mosquito abundance (AaS) by province.** In both analyses, one province, Elías Piña, was excluded because of assumed substantial under-reporting. This province, which has a population of 63,250 according to the 2010 Census, reported 1 dengue case during Season 1 and 1 chikungunya case during that outbreak. (a) Log adjusted attack rates vs AaS. Simple linear regression was used to measure correlation between log cases with AaS. (b) Mean age of infection vs AaS (force of infection = mean age of infection$^{-1}$). Simple linear regression was used to measure correlation between mean age and AaS.

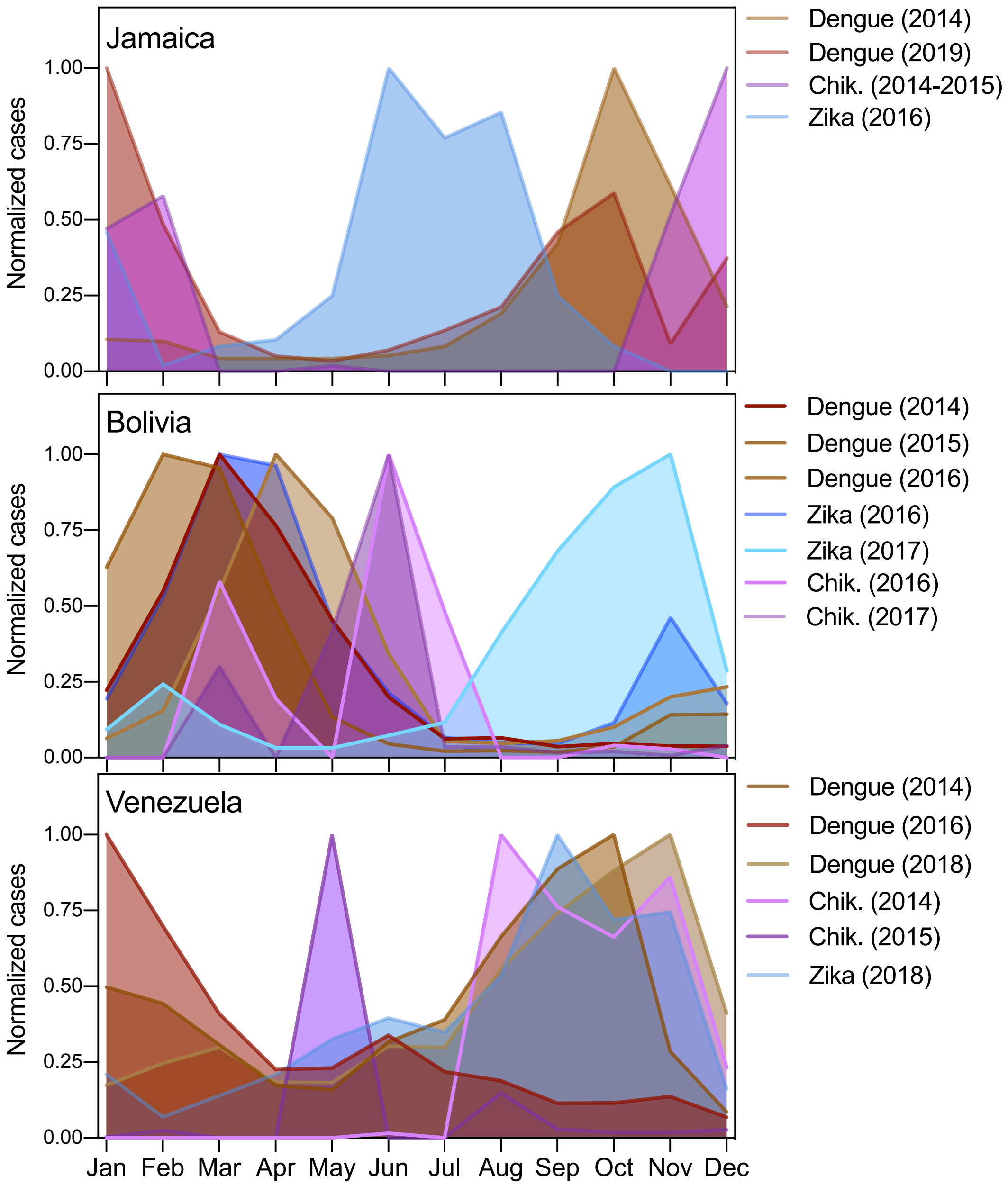


**Supplementary Figure 3: Asynchronicity of reported dengue, chikungunya, and Zika outbreaks in three countries in the Americas.** Cases were normalized by individual outbreak. Monthly (chikungunya) and weekly (dengue and Zika) case data were obtained from the Pan-American Health Organization website (https://www.paho.org/en).

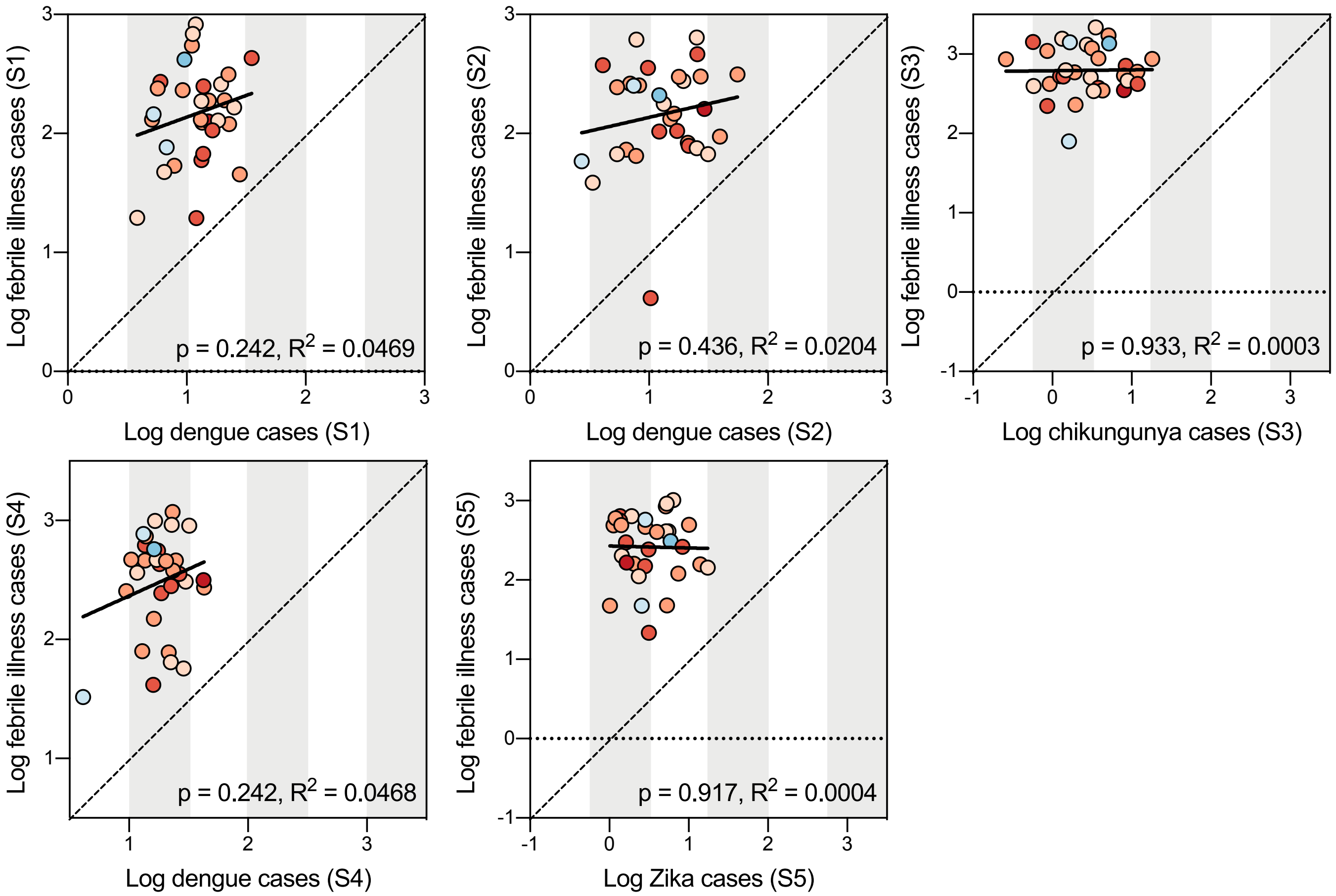


**Supplementary Figure 4: No significant correlation between disease and febrile illness attack rates reported by province during dengue, chikungunya, and Zika outbreaks.** Log population-adjusted attack rates per 10,000 individuals were calculated for each of the 31 provinces using disease and febrile illness data reported during each outbreak. Each data point represents a province. For the reasons stated above, the province Elías Piña was excluded from the analysis. Colors correspond to the scheme used in **Fig. 4c**. Simple linear regression was used to measure correlation between log cases of febrile illness and log cases of dengue, chikungunya, and Zika.

## Supplementary Table 1: Priors for Index P estimates

| Parameter | Prior distribution | Sources |
|---|---|---|
| Human Incubation Period | Gaussian (mean = 5, SD = 1) | 1–3 |
| Human Infectious Period | Gaussian (mean = 5, SD = 1) | 1–3 |
| Human Life Expectancy | Gaussian (mean = 73, SD = 2) | 4 |
| Transmission probability (human to mosquito) | Gaussian (mean = 0.5, SD = 0.01) | 5 |
| Mosquito Biting Rate | Gaussian (mean = 0.25, SD = 0.05) | 6,7 |
| Extrinsic Incubation Period | Gaussian (mean = 7, SD = 2) | 2,8,9 |
| Mosquito Life Expectancy | Gaussian (mean = 14, SD = 3) | 10,11 |

## Supplementary Table 2: $R_{eff}$ estimation parameters

| | **Incubation period (days)** | **Infectious period (days)** | **Serial Interval (days)** | **Sources** |
|---|---|---|---|---|
| Dengue | 4-6 | 9-11 | 13-17 | 12 |
| Zika | 3-14 | 7-8 | 10-23 | 13,14 |
| Chikungunya | 2-12 | 7-9 | 9-21 | 15,16 |

## SUPPLEMENTARY REFERENCES


1. Lourenço, J. *et al.* Epidemiological and ecological determinants of Zika virus transmission in an urban setting. *eLife* vol. 6 (2017).
2. Ferguson, N. M. *et al.* EPIDEMIOLOGY. Countering the Zika epidemic in Latin America. *Science* 353, 353–354 (2016).
3. Lessler, J. *et al.* Times to key events in Zika virus infection and implications for blood donation: a systematic review. *Bull. World Health Organ.* 94, 841–849 (2016).
4. *Dominican Republic: Human Development Indicators*. http://hdr.undp.org/en/countries/profiles/DOM.
5. Obolski, U. *et al.* MVSE : An R-package that estimates a climate-driven mosquito-borne viral suitability index. *Methods Ecol. Evol.* 10, 1357–1370 (2019).
6. Yasuno, M. & Tonn, R. J. A study of biting habits of Aedes aegypti in Bangkok, Thailand. *Bull. World Health Organ.* 43, 319–325 (1970).
7. Trpis, M. & Hausermann, W. Dispersal and other population parameters of Aedes aegypti in an African village and their possible significance in epidemiology of vector-borne diseases. *Am. J. Trop. Med. Hyg.* 35, 1263–1279 (1986).
8. Li, M. I., Wong, P. S. J., Ng, L. C. & Tan, C. H. Oral susceptibility of Singapore Aedes (Stegomyia) aegypti (Linnaeus) to Zika virus. *PLoS Negl. Trop. Dis.* 6, e1792 (2012).
9. Wong, P.-S. J., Li, M.-Z. I., Chong, C.-S., Ng, L.-C. & Tan, C.-H. Aedes (Stegomyia) albopictus (Skuse): a potential vector of Zika virus in Singapore. *PLoS Negl. Trop. Dis.* 7, e2348 (2013).
10. Trpis, M., Häusermann, W. & Craig, G. B. Estimates of Population Size, Dispersal, and Longevity of Domestic Aedes aegypti aegypti (Diptera: Culicidae) by Mark–Release–Recapture in the Village of Shauri Moyo in Eastern Kenya. *Journal of Medical Entomology* vol. 32 27–33 (1995).
11. Hugo, L. E. *et al.* Adult survivorship of the dengue mosquito Aedes aegypti varies seasonally in central Vietnam. *PLoS Negl. Trop. Dis.* 8, e2669 (2014).
12. Aldstadt, J. *et al.* Space-time analysis of hospitalised dengue patients in rural Thailand reveals important temporal intervals in the pattern of dengue virus transmission. *Trop. Med. Int. Health* 17, 1076–1085 (2012).
13. Majumder, M. S., Cohn, E., Fish, D. & Brownstein, J. S. Estimating a feasible serial interval range for Zika fever. *Bull. World Health Organ.* 10, 1–6 (2016).
14. *Zika virus: Fact Sheet*. https://www.who.int/news-room/fact-sheets/detail/zika-virus (2018).
15. Riou, J., Poletto, C. & Boëlle, P.-Y. A comparative analysis of Chikungunya and Zika transmission. *Epidemics* 19, 43–52 (2017).
16. Staples, J. E., Breiman, R. F. & Powers, A. M. Chikungunya fever: an epidemiological review of a re-emerging infectious disease. *Clin. Infect. Dis.* 49, 942–948 (2009).

**Asincronía de los brotes de enfermedades endémicas y emergentes transmitidas por mosquitos en la República Dominicana**

Mary E. Petrone[1‡*], Rebecca Earnest[1‡], José Lourenço[2], Moritz U.G. Kraemer[2], Robert Paulino-Ramirez[3], Nathan D. Grubaugh[1‡], Leandro Tapia[3‡*]

[1]Department of Epidemiology of Microbial Diseases, Yale School of Public Health, New Haven, CT 06510, USA
[2]Department of Zoology, University of Oxford, Oxford, United Kingdom
[3]Instituto de Medicina Tropical & Salud Global, Universidad Iberoamericana, Santo Domingo, Republica Dominicana
‡Estos autores contribuyeron de forma equitativa
Correspondencia a: mary.petrone@yale.edu (M.E.P.); l.tapia@prof.unibe.edu.do (L.T.)

## RESUMEN

Los virus transmitidos por mosquitos amenazan al Caribe debido al clima tropical de la región y la recepción estacional de turistas internacionales. Los brotes de chikungunya y Zika demostraron la rapidez con la que estos virus pueden propagarse. Al mismo tiempo, los casos de dengue han aumentado durante la última década. Se necesitan con urgencia medidas sostenibles de control de enfermedades para sofocar la transmisión del virus y prevenir futuros brotes. Para mejorar los métodos de control actuales, analizamos los patrones temporales y espaciales de los brotes de chikungunya, Zika y dengue notificados en la República Dominicana entre 2012 y 2018. Los virus que causaron estos brotes son transmitidos por los mosquitos Aedes, que son sensibles a la variabilidad climatológica estacional. Aquí, evaluamos si el clima y la dinámica espacio-temporal de los brotes de dengue podrían explicar los patrones de brotes de enfermedades emergentes. Encontramos que los brotes de enfermedades emergentes fueron robustos a las variables de limitaciones climatológicas y espacio-temporales que definen la dinámica de los brotes de dengue estacional, lo que indica que se requiere una vigilancia constante para prevenir futuras crisis de salud.

## INTRODUCCIÓN

Los virus emergentes y endémicos transmitidos por mosquitos son una preocupación constante de salud pública en el Caribe [1–3]. Esta región es especialmente vulnerable a la propagación de la primera debido a su clima tropical y su gran industria turística, que atrae a visitantes de todo el mundo. Los recientes brotes de chikungunya [4–6] y Zika [7–9] en 2014 y 2016, respectivamente, demostraron que los virus que alguna vez estuvieron asociados con enfermedades leves pueden reaparecer y causar resultados de salud devastadores. Los informes de que el virus Mayaro, que ya se ha detectado en el Caribe, puede ser transmitido por la especie de mosquito urbanizado *Aedes*, indican que es posible que se avecinen brotes futuros de enfermedades emergentes transmitidas por mosquitos [10–14]. Además de esta amenaza, el virus del dengue es endémico en muchos países y territorios del Caribe y ha causado brotes con mayor frecuencia durante la última década. Los grandes brotes, que comenzaron en 2019 pero han continuado hasta 2020 [15], extienden una tendencia alarmante de un número creciente de casos de dengue notificados anualmente en las Américas en los últimos años [16]. Sin embargo, el peligro de estos virus y las enfermedades que causan no radica solo en sus síntomas debilitantes y, a veces, potencialmente mortales. La imprevisibilidad de cuándo y dónde ocurrirá un nuevo brote impide la preparación. Las estrategias de respuesta a los brotes son intrínsecamente reaccionarias y, en su transitoriedad, perturban los sistemas de salud pública cuando se introducen nuevas iniciativas y posteriormente se eliminan. Esto es especialmente problemático en entornos con recursos limitados donde se debe priorizar la asignación estratégica de recursos para maximizar el impacto de los esfuerzos de control de enfermedades. Se necesitan nuevos enfoques centrados en la vigilancia sostenible a largo plazo para reducir el potencial de futuras crisis de salud pública en el Caribe.

Uno de esos enfoques es el uso de datos climáticos para evaluar el riesgo de propagación viral. La transmisión de virus transmitidos por mosquitos, tanto emergentes como endémicos, debe adherirse a patrones temporales y espaciales similares de manera que la dinámica de brotes pasados pueda informar a los de brotes futuros. Muchos flavivirus, como los virus del dengue y Zika, y los alfavirus, como el chikungunya, son transmitidos por mosquitos *Aedes*, que son sensibles a la variabilidad climatológica [17–19]. Se ha documentado la concordancia temporal entre los patrones climáticos estacionales y la transmisión del virus de la fiebre amarilla (flavivirus) en el continente africano [20]. Se han reportado asociaciones similares para la incidencia de la fiebre del dengue en Hanoi, Vietnam y Myanmar [21,22] y para la incidencia de chikungunya y Zika en las Américas [19]. Sin embargo, existe evidencia que sugiere que esta relación no se extiende a la dinámica de transmisión de virus durante su primer año de introducción en una nueva población [7,23]. Por lo tanto, consideramos si, si bien el clima puede ser un indicador útil para futuros brotes de virus endémicos, se deben considerar otros factores, incluido el tamaño de la población, la demografía y el momento de la introducción, al desarrollar estrategias para prevenir futuros brotes de enfermedades emergentes.

Para responder a estas preguntas en el contexto del Caribe, analizamos los casos de dengue, chikungunya y Zika reportados diariamente en la República Dominicana entre 2012 y 2018. Encontramos que los brotes de enfermedades emergentes (chikungunya y Zika) ocurrieron antes en el año que los brotes de dengue y el momento y la ubicación de las introducciones de virus emergentes impactados cuándo y dónde ocurrieron los brotes correspondientes. Además, la propagación de los virus chikungunya y Zika fue tolerante a climas subóptimos para la transmisión por mosquitos *Aedes*, probablemente debido al gran tamaño de la población humana susceptible. La abundancia pronosticada de mosquitos fue igualmente poco informativa para la distribución espacial de las tasas de ataque de enfermedades emergentes y la fuerza de la infección. Además, las tasas de ataque de dengue a nivel provincial fueron consistentes entre los brotes de dengue, pero no se correspondieron con las tasas de ataque local de chikungunya y Zika. En conjunto, demostramos que el virus del dengue puede no ser siempre un modelo apropiado para prepararse para futuros brotes emergentes de enfermedades transmitidas por mosquitos. En cambio, se necesita un sistema de vigilancia de enfermedades transmitidas por mosquitos sostenible y a largo plazo para facilitar respuestas proactivas a las amenazas emergentes y para rastrear la propagación continua de enfermedades conocidas como el dengue, la chikungunya y el Zika. Proponemos específicamente el uso de dos indicadores, la incidencia de casos de enfermedades febriles y las tasas de letalidad por dengue, para monitorear el desempeño de la vigilancia e identificar posibles amenazas emergentes.

## RESULTADOS

### Múltiples brotes endémicos y emergentes de virus transmitidos por mosquitos en República Dominicana

Entre 2012 y 2018, República Dominicana, un país que comparte la isla caribeña La Hispaniola con Haití (Fig.1a), experimentó cinco brotes de enfermedades causadas por virus transmitidos por mosquitos (Fig.1b, c). Delimitamos este período en seis temporadas (Temporadas 1-6), cada una a partir de abril de cada año entre 2012 y 2018, coincidiendo con el inicio de la temporada de lluvias. Tres de los cinco brotes fueron causados por el virus del dengue (Temporadas 1, 2 y 4; Fig. 1b, c). No se dispone de suficientes datos de la encuesta serológica para la República Dominicana para determinar el (los) serotipo (s) predominante (s) del virus del dengue durante cada brote, pero la Organización Panamericana de la Salud informa que los serotipos 1, 2 y 4 prevalecieron en el Caribe entre 2012 y 2014; y que los serotipos 2, 3 y 4 estaban circulando en la región en 2015 [24]. Los otros dos brotes fueron causados por virus emergentes, chikungunya y Zika (temporadas 3 y 5, respectivamente; Fig.1b, c) [25]. Esos brotes fueron los primeros y únicos en notificarse de cualquiera de las enfermedades en el país, y el número de casos notificados en el Caribe en su conjunto se desplomó en los años siguientes [26,27]. Después de la temporada dominada por el virus Zika (temporada 5), solo se diagnosticó una pequeña cantidad de casos de dengue (temporada 6).

Las características clínicas y demográficas difirieron entre los casos notificados de dengue, chikungunya y Zika. La mayoría de los casos de dengue presentaron fiebre (98,3%), fueron hospitalizados por su condición (93,7%) y tenían entre 0 y 15 años (62,3%), lo que sugiere inmunidad en la población adulta (Cuadro 1). Los pacientes diagnosticados con chikungunya y Zika eran mayores y predominantemente mujeres. El sarpullido y / o la artralgia junto con la fiebre son manifestaciones clínicas típicas de las infecciones por chikungunya y Zika [28–33]. Aunque más bajas que las tasas reportadas en otros lugares, las tasas de artralgia entre los casos de chikungunya en nuestro conjunto de datos fueron consistentes con las tasas documentadas previamente entre las personas diagnosticadas con chikungunya en la República Dominicana [30].

Tabla 1: Características epidemiológicas y clínicas de los casos

| Temporada y Enfermedad | Año | Número de Casos | Edad Media de los Casos (IQR) | % Hospitalizados | % Femenino | % Fiebre Reportada | % Rash Reportado | % Artralgia Reportada |
|---|---|---|---|---|---|---|---|---|
| Temporada 1 (dengue)* | 2012-2013 | 13,666 | 11 (11) | 94.3 | 44.0 | 98.7 | 15.0 | 31.0 |
| Temporada 2 (dengue)* | 2013-2014 | 15,079 | 11 (11) | 92.9 | 46.3 | 97.4 | 5.6 | 16.4 |
| Temporada 3 (dengue) | 2014-2015 | 5,236 | 13 (18) | 88.7 | 45.0 | 96.1 | 8.0 | 23.2 |
| Temporada 4 (dengue)* | 2015-2016 | 19,619 | 12 (13) | 93.9 | 46.0 | 98.9 | 4.0 | 17.6 |
| Temporada 5 (dengue) | 2016-2017 | 3,093 | 15 (25) | 85.5 | 42.4 | 98.1 | 7.1 | 24.9 |
| Temporada 6 (dengue) | 2017-2018 | 1,437 | 18 (24) | 87.3** | 38.5 | 98.3 | 4.0 | 27.4 |
| Chikungunya | 2014 | 6,461 | 28 (32) | 32.0 | 60.8 | 89.5 | 26.0 | 63.0 |
| Zika | 2016 | 5,161 | 30 (21) | 16.1 | 73.9 | 74.2 | 70.7 | 8.5 |

IQR: rango intercuartílico
* Brote informado
** El tipo de atención médica que recibió el 22,9% de los casos reportados durante este período no fue reportado y se asumió como ingresos hospitalarios.

## La dinámica de los brotes de chikungunya y Zika no se ajusta a los patrones estacionales del dengue

La implementación de medidas efectivas de prevención y control de enfermedades requiere conocer cuándo es más probable que sus virus etiológicos emerjan, reaparezcan y se propaguen [34] . El dengue, el chikungunya y el Zika son causados por virus ARN transmitidos por los mosquitos vectores *Aedes aegypti* y *Aedes albopictus*. La abundancia y la capacidad de estos vectores son sensibles a factores climatológicos como la temperatura, la lluvia y la humedad [17,18], de modo que se ha demostrado que la transmisión de los tres virus fluctúa en especie [19–22,35]. Por lo tanto, planteamos la hipótesis de que el momento de los cinco brotes debería concordar con los patrones climáticos estacionales.

Encontramos que los brotes de enfermedades emergentes (chikungunya [Temporada 3] y Zika [Temporada 5]) ocurrieron a principios de año en comparación con los brotes endémicos (dengue). Los brotes de chikungunya y Zika alcanzaron su punto máximo 15 y 26 semanas antes, respectivamente, que el pico de dengue promedio (semana epidemiológica 41; Fig. 1b). Si bien los casos de chikungunya y dengue comenzaron a aumentar alrededor de la semana epidemiológica 15, se observó un aumento en los casos de Zika durante las primeras semanas del año.

Debido a que la implementación de la notificación a nivel nacional de casos de chikungunya y Zika influyó en el momento en que se detectaron estos brotes (Figura 1c, flechas), calculamos el número de semanas transcurridas entre el primer caso notificado de cada brote y el número máximo de casos dentro de las provincias (Figura 1d). Supusimos que los factores climatológicos no variaban mucho entre provincias durante una temporada

determinada. Nuestro razonamiento se basa en que la tasa de transmisión viral está limitada por el período de incubación extrínseco (EIP) del virus en el mosquito vector [36], y el EIP para los mosquitos *Aedes* está influenciado principalmente por la temperatura [37–39]. Por lo tanto, debido a que no esperábamos que las tasas de transmisión variaran mucho entre las provincias, los brotes provinciales que alcanzaron su punto máximo poco después de la implementación del sistema de notificación indicarían que la transmisión en esas provincias precedió al establecimiento de este sistema.

Nuestro análisis identificó uno de esos casos. La mayoría de los brotes provinciales de chikungunya y Zika alcanzaron su punto máximo dentro de las 12 y 25 semanas, y el tiempo medio para alcanzar el pico en las provincias no difirió significativamente (Fig. 1d, e). Esto contrastaba con la heterogeneidad sustancial del momento de los tres brotes de dengue. La falta de uniformidad entre estos brotes puede deberse a una tasa de propagación más lenta entre las regiones debido a un panorama de inmunidad colectiva preexistente y espacialmente heterogéneo. Cinco provincias, cuatro de las cuales están ubicadas en la parte suroeste del país, reportaron números máximos de casos de Zika en enero y febrero de 2016 (Fig. 1d). Por las razones expuestas anteriormente y debido a que el virus del Zika había estado circulando en las Américas durante al menos dos años antes de que se informara en la República Dominicana [7,40], esta observación sugirió que el virus del Zika ya estaba circulando en esas provincias antes de que se implementara el sistema nacional de presentación de casos obligatorios a principios de enero. Planteamos la hipótesis de que la transmisión temprana del virus del Zika podría haber ocurrido en la región suroeste debido a la proximidad de la región a Haití. En particular, esta región comparte frontera con Haití, está conectada con la capital de Haití, Puerto Príncipe, a través de una carretera principal y alberga un gran mercado binacional (Fig. 1e, mapa, estrella). Una comparación temporal de los brotes de Zika reportados en esta región, el resto del país y Haití reveló que el brote regional fue más consistente con el brote de Zika de 2016 informado en Haití (Figura 1e, recuadro). Además, la proporción de casos de dengue que informaron una erupción fue mayor y alcanzó su punto máximo antes en la región suroeste de Enriquillo en 2016 en comparación con el resto del país (Figura Suplementaria 1). Esto sugiere que algunos casos de Zika pueden haber sido clasificados erróneamente como dengue al comienzo del brote. Estos hallazgos indican que la República Dominicana experimentó dos brotes de Zika geográfica y temporalmente distintos. Solo a partir de los recuentos de casos notificados, no podemos concluir si el virus del Zika se introdujo en la República Dominicana desde Haití o viceversa; sin embargo, está claro que la coordinación binacional es un componente esencial del control local de enfermedades transmitidas por mosquitos porque los virus que transmiten estos vectores no reconocen fronteras internacionales.

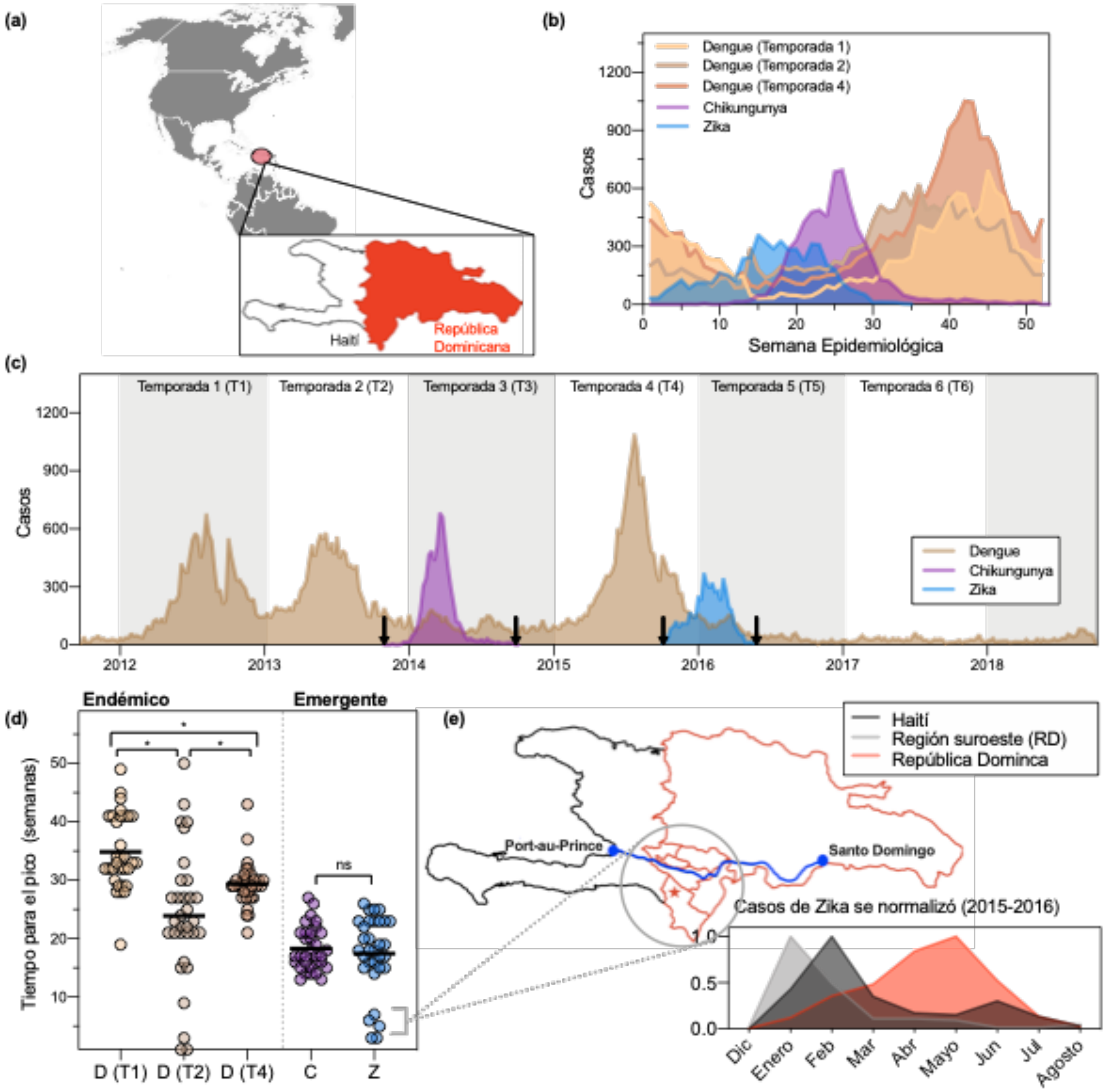


**Figura 1: Se notificaron dos brotes de enfermedades emergentes, chikungunya y Zika, y tres brotes endémicos de dengue entre 2012 y 2018 en la República Dominicana.** (**a**) La República Dominicana es un país del Caribe que comparte la isla de La Hispaniola con Haití. Mapa: *Free Vector Maps* (**b**) Casos en República Dominicana notificados por el Ministerio de Salud durante cada brote por semana epidemiológica durante cinco temporadas. (**c**) Casos notificados semanalmente de 2012 a 2018. Las flechas indican cuándo se notificaron el primer y último caso de patógenos emergentes. (**d**) Número de semanas transcurridas entre el primer caso notificado a nivel nacional y los casos máximos notificados en 32 provincias (cada punto de datos representa una provincia diferente). El brote de chikungunya comenzó durante la semana epidemiológica (SE) 6, el brote de Zika durante la SE 1 y los tres brotes de dengue durante la SE 14. Se comparó el tiempo medio hasta el pico de cada brote mediante ANOVA. El resultado estadístico cualitativo del análisis no cambió cuando se excluyeron las cinco provincias tempranas del Zika. D(T1) vs D(T2): $p < 0.0001$; D(T1) vs D(T4): $p = 0.0155$; D(T2) vs D(T4): $p = 0.0173$; C vs Z: $p = 0.9885$ (**e**) Comparaciones geográficas y temporales de los casos de Zika notificados por cuatro provincias de la región suroeste de la República Dominicana. Una carretera principal conecta la capital de Haití, Puerto Príncipe, con la región suroeste de la República Dominicana (línea azul, mapa) y Santo Domingo, la capital nacional. Un gran mercado binacional se ubica en la provincia de Pedernales (estrella, mapa). Al igual que en Haití, las provincias del suroeste experimentaron un brote anterior de Zika en comparación con las 28 provincias restantes (recuadro).

**Los brotes iniciales de enfermedades emergentes transmitidas por mosquitos pueden ocurrir durante períodos de condiciones climatológicas subóptimas**

Habiendo observado que los brotes de enfermedades emergentes alcanzaron su punto máximo a principios de año que los tres brotes de dengue (Fig. 1), consideramos dos posibles explicaciones: (1) O los patrones climáticos diferían entre las estaciones y los brotes emergentes precedieron a los del dengue debido a la estocasticidad estacional, o (2) la propagación de los virus emergentes fue menos sensible a los factores climatológicos en comparación con el virus del dengue. Al analizar los datos climatológicos diarios recopilados durante la duración de nuestro período de estudio, encontramos apoyo para este último (Fig. 2).

Para investigar la relación entre el clima y la incidencia de casos, utilizamos datos de series de tiempo de temperatura y humedad para estimar el potencial de transmisión a través de mosquitos durante nuestro período de estudio (Fig. 2). Para este análisis, utilizamos el Índice P, una métrica que se calcula incorporando datos climáticos y entomológicos en un marco bayesiano para estimar el potencial de transmisión de mosquitos hembra individuales [19]. Calculamos el potencial de transmisión para cada semana de nuestro período de estudio utilizando datos reportados en seis de treinta y dos provincias. Seleccionamos estas seis provincias en función de su representatividad de los subclimas del país y la disponibilidad de datos. En promedio, el potencial de transmisión fluctuó estacionalmente, aumentando entre abril y noviembre de cada año, coincidiendo con la temporada de lluvias, y disminuyó poco después (Fig. 2). Cuando comparamos la dinámica temporal del potencial de transmisión con la incidencia de la enfermedad notificada mediante inspección visual, encontramos que el número de casos de enfermedades emergentes notificados semanalmente alcanzó su punto máximo antes de que el potencial de transmisión hubiera alcanzado un máximo estacional para ambos brotes (chikungunya y Zika), mientras que el número de Los casos de dengue notificados semanalmente alcanzaron su punto máximo después de este punto en dos de los tres brotes de dengue (Fig. 2a). Medimos el número de semanas transcurridas entre los casos de brotes y el Índice P durante cada temporada y descubrimos que, en promedio, los casos de dengue alcanzaron su punto máximo 17 semanas después del Índice P. En contraste, los casos de chikungunya alcanzaron su punto máximo 14 semanas y los casos de Zika alcanzaron su punto máximo 27 semanas antes que el Índice P (Fig. 2a, círculos). Persistieron patrones climatológicos similares a nivel provincial (Fig. 2b). Algunas provincias experimentaron claras fluctuaciones estacionales en el potencial de transmisión, mientras que los picos y valles estacionales fueron menos definidos en el Distrito Nacional y María Trinidad Sánchez, que tienen subclimas de baja a moderada humedad. A pesar de esta variación, el momento de los brotes provinciales se ajustó a la tendencia observada con el examen visual a nivel nacional: los brotes de dengue alcanzaron su punto máximo después de un período caracterizado por un alto potencial de transmisión, justo cuando el potencial de transmisión comenzaba a disminuir; por el contrario, los brotes de enfermedades emergentes ocurrieron al mismo tiempo que aumentaba el potencial de transmisión. Además, no podemos atribuir las anomalías climáticas a la disminución de los casos de dengue notificados en las temporadas 3, 5 y 6.

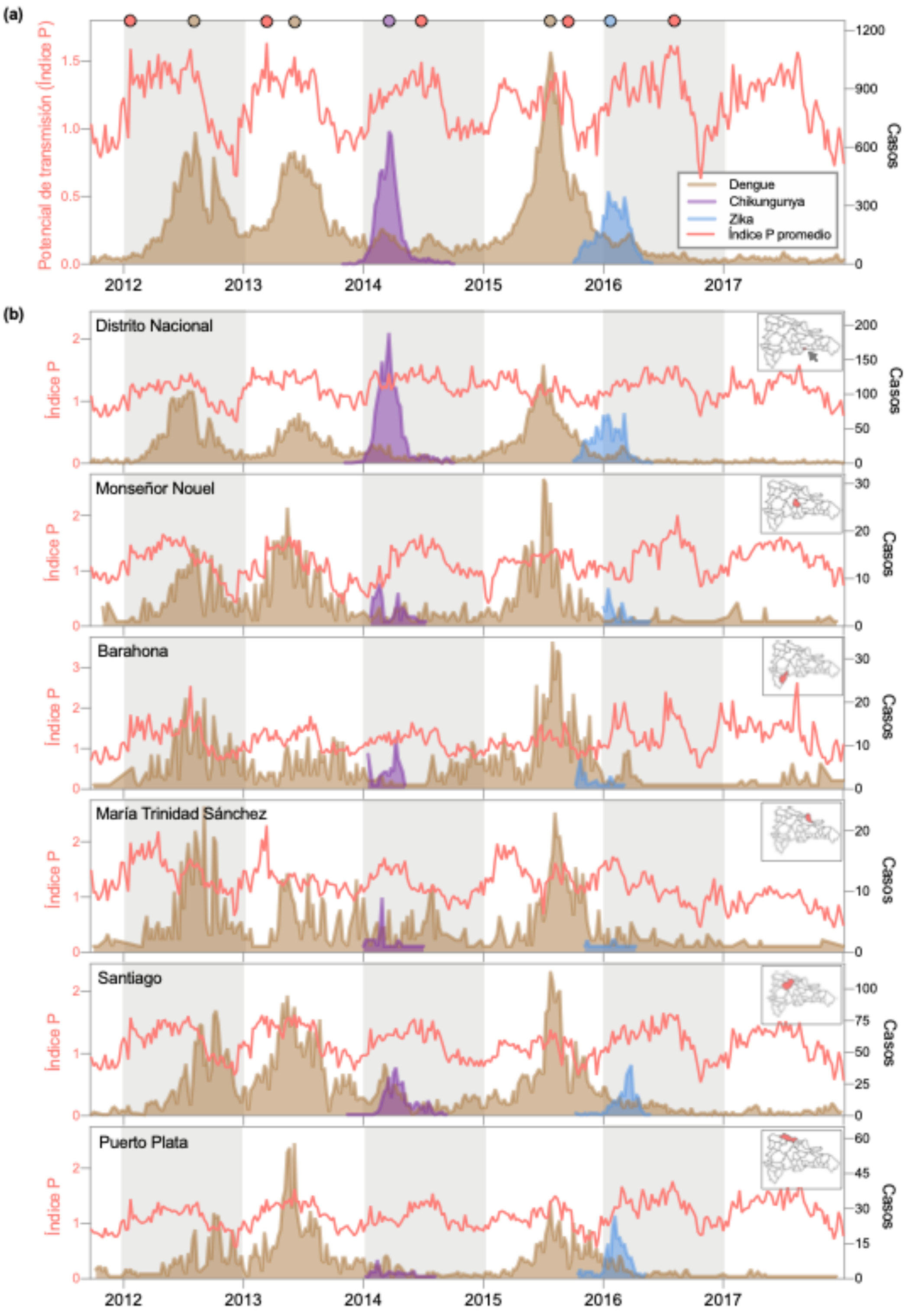


**Figura 2**. **Los brotes de enfermedades emergentes ocurrieron durante períodos de clima subóptimo.** (a) Potencial de transmisión semanal promedio (Índice P) y casos notificados a nivel nacional. El índice P describe el potencial reproductivo de un mosquito Aedes hembra individual, lo que significa que su valor absoluto debe interpretarse biológicamente más que epidemiológicamente. Las fluctuaciones relativas en el índice P reflejan cambios estacionales en la tasa esperada de transmisión de virus transmitidos por mosquitos. Los picos de cada curva durante cada temporada se indican como círculos en el eje x superior. (b) Índice P y casos notificados para seis provincias. Los datos de temperatura y humedad se obtuvieron de la base de datos de la Oficina Nacional de Meteorología (ONAMET) (saip.gob.do) para el Distrito Nacional, Barahona, Puerto Plata, María Trinidad Sánchez y Santiago. Los datos climatológicos horarios de Monseñor Nouel se obtuvieron de Open Weather Map. Los casos notificados (openweathermap.org) de dengue, chikungunya y Zika se organizaron por provincia según el lugar de residencia del individuo.

**El tamaño de la población humana susceptible influyó en la velocidad a la que se propagan los virus emergentes.**

La variabilidad en los patrones climáticos estacionales y la capacidad vectorial no explicaron las diferencias en el momento de los brotes de enfermedades emergentes (Fig. 2). Más bien, planteamos la hipótesis de que los patrones observados para el dengue probablemente hayan sido influenciados por un panorama de inmunidad de manada preexistente y espacialmente heterogéneo. Esto obligaría a que el potencial de transmisión permanezca alto durante un período prolongado antes de que se logre el crecimiento epidémico cada temporada.

Para probar la hipótesis de que la susceptibilidad local influyó en el momento de los brotes, examinamos la relación entre el tamaño de la población humana susceptible y el tiempo de duplicación de cada brote (Fig. 3). Estimamos que la mediana del número de reproducción efectiva estimada ($R_{eff}$) para los tres brotes de dengue estuvo entre 1.3 y 1.4, mientras que los de los brotes de chikungunya y Zika variaron de 1.6 a 2.45 (Fig. 3a). Estos hallazgos son consistentes con la epidemiología de las tres enfermedades y estimaciones previas de $R_{eff}$ para chikungunya y Zika [25,41,42]. Las estimaciones publicadas del número de reproducción básico (R0) para el dengue, equivalente a Reff en una población totalmente susceptible, varían ampliamente (0,97-65) debido a la sensibilidad del parámetro al tamaño de la población local susceptible [43]. Observamos un tiempo de duplicación un poco más largo (menor $R_{eff}$) durante el tercer brote de dengue, consistente con el aumento de los niveles de inmunidad colectiva de los dos brotes anteriores. En nuestro conjunto de datos, los casos de dengue notificados se produjeron principalmente en el grupo de 0 a 15 años, lo que indica que los grupos de mayor edad tenían niveles altos de inmunidad preexistente y no eran susceptibles a la enfermedad (Tabla 1; Fig. 3b). Por el contrario, especulamos que toda la población era susceptible al chikungunya y al Zika, lo que permitió que estos virus se propagaran a pesar de las condiciones climáticas subóptimas, facilitando un pico de brote más temprano y afectando un rango de edades mucho más amplio (Tabla 1; Fig. 3b).

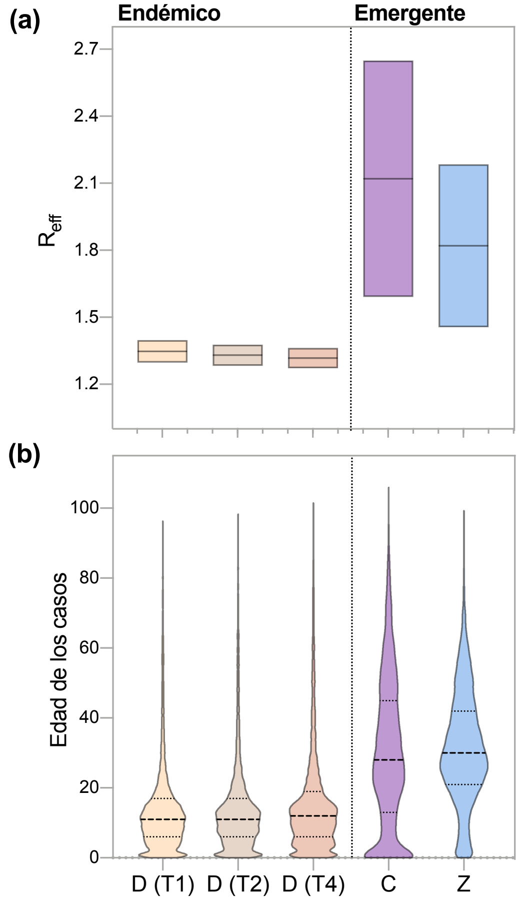


**Figura 3.** El tamaño de la población susceptible a las enfermedades emergentes fue mayor que el dengue. (a) El número de reproducción efectiva ($R_{eff}$) de chikungunya y Zika superó con creces al del dengue. $R_{eff}$ se calculó utilizando la tasa de crecimiento de cada brote [44] y estimaciones del período de incubación y el intervalo de serie de cada enfermedad (Tabla Suplementaria 2). (b) La edad promedio de los casos notificados durante los brotes de enfermedades emergentes superó significativamente la de los tres brotes de dengue. El grupo de edad promedio de la población nacional es de 20 a 25 años, según se calcula a partir de los datos de población informados en el censo de 2010.

## La distribución espacial de las tasas de ataque de dengue es un mal indicador de los brotes iniciales de enfermedades emergentes

Dado que el momento de los brotes de enfermedades emergentes (chikungunya y Zika) no se ajustaba al de los brotes de dengue (Fig. 1,2), sospechamos que la distribución espacial de los casos de dengue sería igualmente poco informativa para los brotes de chikungunya y Zika. Específicamente, planteamos la hipótesis de que la carga relativa del dengue dentro de las provincias durante los brotes individuales se correlacionaría con la carga del dengue durante los brotes posteriores, pero no con la carga relativa de los casos de enfermedades emergentes.

Para abordar esta pregunta, medimos las tasas de ataque ajustadas por edad y sexo por provincia para cada uno de los brotes. Encontramos que las tasas de ataque para las provincias individuales a través de los brotes estaban bien correlacionadas entre los brotes de dengue y entre los brotes de chikungunya y Zika, pero no entre los virus endémicos y emergentes (Fig. 4a, b).

A continuación, investigamos el papel del clima y el uso de la tierra en la perpetuación de esta tendencia, razonando que poblaciones más grandes de mosquitos facilitarían tasas de ataque más altas [18,45]. Con este fin, comparamos un puntaje de idoneidad de *Aedes aegypti* (AaS; Fig.4c), una métrica que incorpora variables ecológicas no incluidas en nuestras estimaciones del potencial de transmisión, como los niveles de vegetación y la precipitación, con las tasas de ataque de la enfermedad ajustadas a la población (Fig. Suplementaria 2, figura

4a). Además, a diferencia de nuestras estimaciones de potencial de transmisión, AaS es una estimación agregada de idoneidad por mes en un año determinado. Curiosamente, el AaS no se correlacionó bien con las tasas de ataque provinciales ajustadas o con la edad media de infección (Figura Suplementaria 2). Además, las provincias en las que se pronosticaron los niveles más altos de abundancia de mosquitos no informaron consistentemente la carga más alta de enfermedad (Fig. 4a, c). Una razón de estas inconsistencias es que AaS se basa en datos climáticos históricos de 1970 a 2000 para estimar la abundancia de mosquitos. Por lo tanto, nuestras estimaciones no capturan eventos climatológicos anómalos como el huracán que tocó tierra en la República Dominicana en octubre de 2012. AaS también asume un alto nivel de estabilidad en el uso del suelo y una urbanización mínima desde el período de treinta años a partir del cual se realizaron las estimaciones. Independientemente de la verdadera razón subyacente de estas discrepancias, las distribuciones espaciales entre las enfermedades endémicas (dengue) y emergentes (chikungunya y Zika) transmitidas por mosquitos no están muy bien correlacionadas.

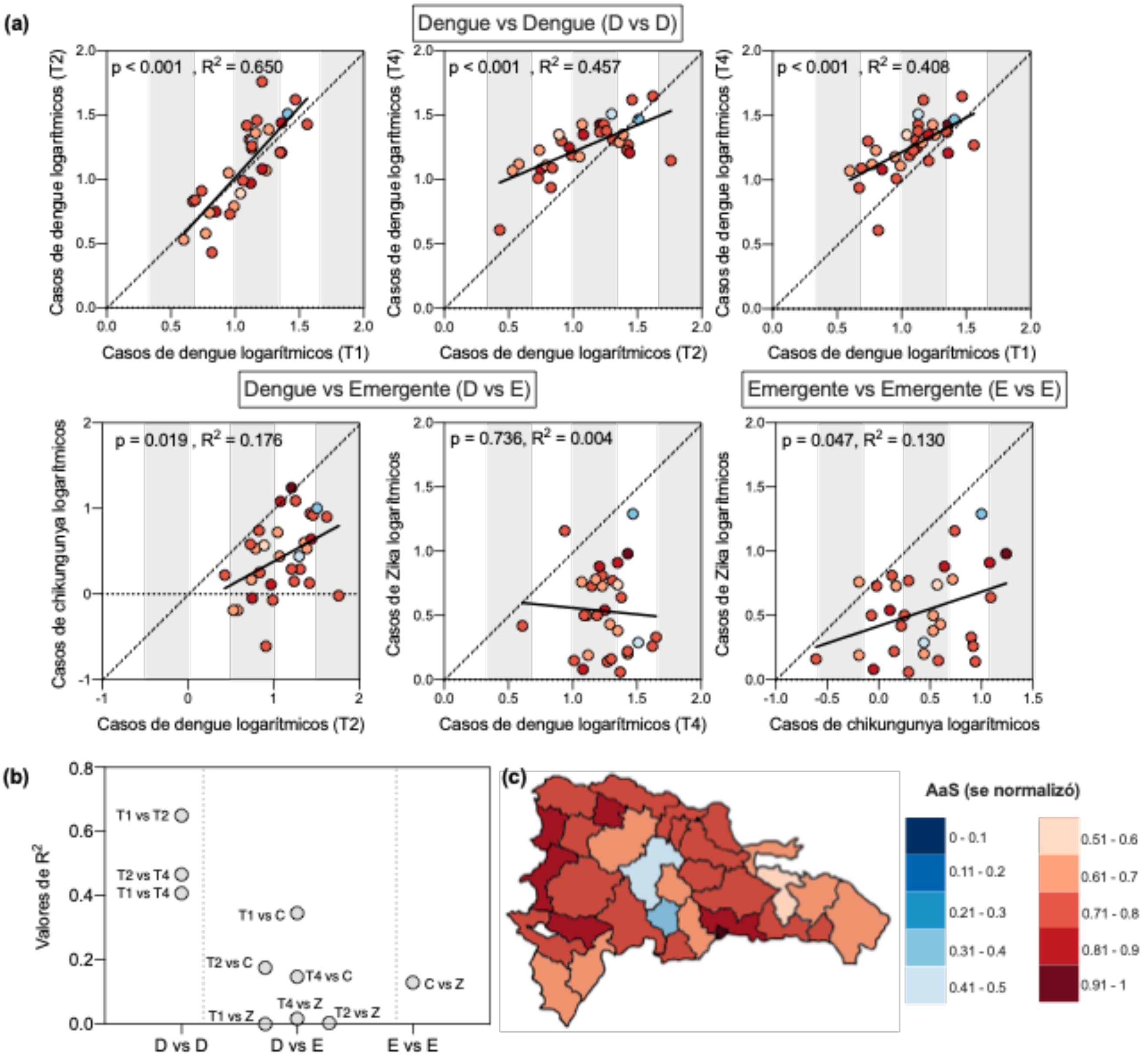


**Figura 4: La distribución espacial de los casos de dengue no está bien correlacionada con la de los casos de enfermedades emergentes o con la abundancia estimada de mosquitos.** (a) Las tasas de ataque ajustadas por población, edad y sexo se correlacionaron significativamente dentro de las provincias entre los brotes de dengue y entre los brotes de Zika y chikungunya. Una provincia, Elías Piña, fue excluida debido a la supuesta

subnotificación sustancial: la provincia notificó 1 caso de dengue durante la temporada 1 y 1 caso de chikungunya en una población de 63.250. (b) Valores de R2 para dengue vs dengue (D vs D), dengue vs emergente (D vs E) y emergente vs emergente (E vs E) calculados por regresión lineal. (c) La abundancia de mosquitos (AaS) se normalizó en todas las provincias.

### Los resultados clínicos de los casos de dengue y las tendencias en los casos de enfermedad febril son indicadores de la prevalencia de la enfermedad

Hasta este punto, nuestros hallazgos demuestran que los brotes emergentes de enfermedades transmitidas por mosquitos no se limitan a los patrones temporales y espaciales de los brotes de enfermedades endémicas en la República Dominicana (Figs. 1-4). Si bien se han observado inconsistencias similares en la dinámica de los brotes en otras partes de las Américas (Figura Suplementaria 3) [23,35], es difícil discernir si nuestros hallazgos se deben a diferencias en la epidemiología o en sesgos de notificación subyacentes. Por lo tanto, utilizamos patrones generales de enfermedad febril y características clínicas para mostrar que los sesgos de notificación no influyeron en nuestras conclusiones previas (Fig. 5).

Debido a que la gran mayoría de los casos en nuestro conjunto de datos informaron fiebre independientemente de la enfermedad (Tabla 1), planteamos la hipótesis de que la incidencia de la enfermedad febril debería reflejar la incidencia de la enfermedad informada durante el período de nuestro estudio. Cuando examinamos el número de casos de enfermedad febril notificados en la República Dominicana por semana epidemiológica por temporada, encontramos que las tendencias temporales en los casos de enfermedad febril en la Temporada 3 (2014-2015) y la Temporada 5 (2016-2017; Figura 5a) fueron consistentes con los que observamos en nuestros datos de casos de chikungunya y Zika, respectivamente (Fig. 1a). En la temporada 4 (2015-2016, dengue), los casos de enfermedad febril alcanzaron su punto máximo en la semana 44, coincidiendo con el pico del brote de dengue (Fig. 1a). Para asegurarnos de que las relaciones espaciales que identificamos en la Figura 4 no fueran puramente el producto de prácticas de notificación diferenciales, comparamos las tasas de ataque ajustadas por población de enfermedades transmitidas por mosquitos y enfermedades febriles notificadas por provincias individuales durante cada temporada y no encontramos una correlación significativa (Figura Suplementaria 4). No se observaron picos estacionales igualmente pronunciados de enfermedad febril informada en la Temporada 1 y la Temporada 2 a pesar de los brotes de dengue conocidos que ocurrieron dentro de ese período. Especulamos que los cambios realizados en los protocolos de notificación de casos de enfermedad febril durante el brote de chikungunya en la temporada 3 provocaron un aumento sostenido en la notificación nacional a partir de entonces [46].

Luego investigamos por qué los casos de enfermedades febriles reportados alcanzaron su punto máximo estacional en las Temporadas 5 y 6 a pesar de una aparente disminución en los casos de dengue coincidentes al comparar las tasas de letalidad (CFR) de los casos de dengue en las estaciones. Específicamente, consideramos la posibilidad de que esta disminución se deba a la subnotificación de casos de dengue durante los períodos intermedios entre los brotes de dengue (temporadas 3, 5 y 6). El CFR promedio durante estas temporadas fue 1.87% (rango: 1.60% -2.40%), mientras que el CFR promedio de los casos reportados durante los tres grandes brotes de dengue en las Temporadas 1, 2 y 4 fue 0.8% (rango: 0.48% -1.20 %) (Figura 5b). En la literatura se informa una amplia gama de CFR del dengue, con una media de 1,62% (IC del 95%: 0,64 a 4,02%) [47], y sin datos de vigilancia serológica, no podemos descartar la posibilidad que la circulación de diferentes serotipos de dengue resultó en diferencias en las CFR estacionales. Sin embargo, si asumimos que la mortalidad entre los casos durante el tercer brote de dengue (temporada 4) se informó correctamente, los casos de dengue se notificaron menos en un 33% en la temporada 3 y en la temporada 5, y en el doble en la temporada 6. estimaciones conservadoras ya que el sistema de vigilancia actual es pasivo y, por lo tanto, no captura los casos asintomáticos. Como resultado, las Temporadas 3, 5 y 6 pueden representar períodos caracterizados por una gran cantidad de casos leves que no fueron capturados por el sistema de vigilancia. También es probable que el número de muertes debidas al chikungunya no se haya informado sustancialmente; sin embargo, nuestro cálculo de 0.19% corresponde a una estimación post-hoc corregida de esta tasa (0.15%) para República Dominicana [48].

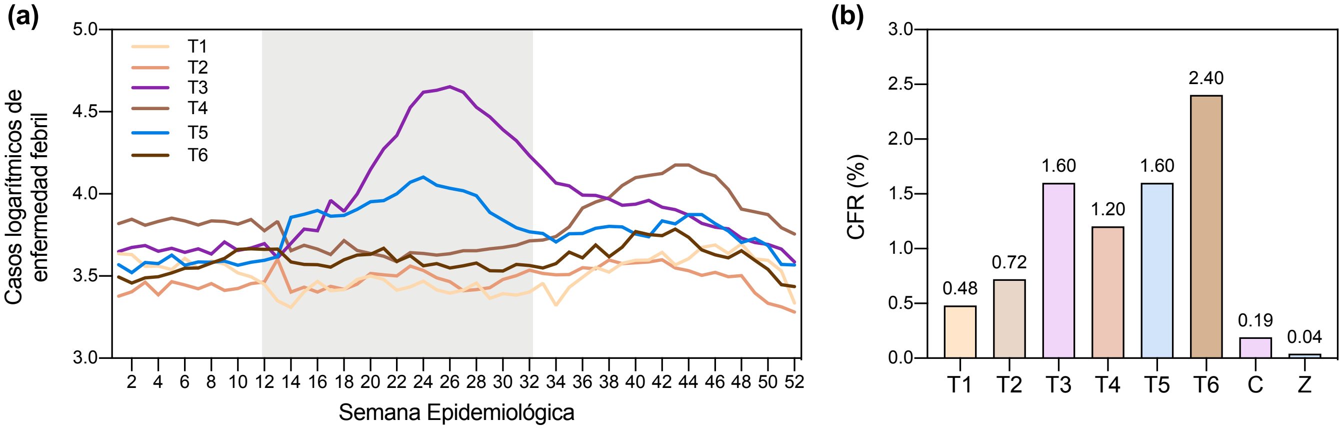


**Figura 5.** Los casos notificados de enfermedad febril y las tasas de letalidad de casos de dengue durante los períodos entre brotes de dengue superaron a los notificados durante los brotes de dengue. (a) Las tendencias temporales en los casos de enfermedades febriles semanales reflejan las tendencias temporales en los casos notificados de dengue, chikungunya y zika. Los casos de enfermedad febril aguda reportados por semana epidemiológica por municipio se obtuvieron de la Dirección Nacional de Estadísticas del Ministerio de Salud (saip.gob.do). Los casos semanales se agregaron a nivel nacional. Los picos de los brotes de chikungunya y zika se indican con el cuadro gris. (b) Las tasas de letalidad porcentual (CFR) de los casos de dengue aumentaron durante las temporadas en las que no se notificó ningún brote de dengue. Los CFR se calcularon para seis temporadas de casos notificados de dengue (T1-T6) y dos temporadas de casos de enfermedades emergentes (chikungunya (C) y Zika (Z)).

## DISCUSIÓN

Nuestro estudio demuestra que, incluso cuando se transmiten por el mismo mosquito vector, los virus no están sujetos a la misma dinámica temporal y espacial de los brotes. En cambio, cuándo y dónde se introduce el nuevo virus, el tamaño de la población humana susceptible y la capacidad de los sistemas de vigilancia locales determinan que se detecten estas dinámicas. En resumen, la epidemiología del dengue no puede utilizarse para anticipar la ubicación y el momento de los brotes futuros de enfermedades emergentes transmitidas por mosquitos en la República Dominicana y probablemente en otros países y territorios del Caribe. En cambio, se deben implementar métodos de vigilancia consistentes y sostenibles para limitar las enfermedades y prevenir brotes futuros. Estos métodos podrían incluir la vigilancia serológica de la población durante los períodos entre los brotes [49], la prueba de los vectores de mosquitos locales para detectar infecciones virales [50–52] y el monitoreo de los resultados de salud de viajeros que visitan el país [53].

Mantener un sistema de vigilancia sostenible es fundamental para prevenir la transmisión silenciosa de virus que pueden alimentar grandes brotes. Otros países de las Américas informaron sobre brotes posteriores de chikungunya y Zika después de sus brotes iniciales. No podemos determinar de manera concluyente si la República Dominicana experimentó un patrón similar porque los datos de vigilancia de chikungunya y Zika no están disponibles para las temporadas posteriores a los brotes iniciales de estas enfermedades. Elucidar si el dengue, el chikungunya y el Zika están co-circulando en el país será fundamental para clasificar y brindar la atención clínica adecuada a los pacientes que presentan una enfermedad febril [54], especialmente si la transmisión del virus del chikungunya y el Zika es ahora. en sincronía con la transmisión del dengue [55,56]. Comprender el papel de la inmunidad en la modulación de la tasa de propagación de arbovirus en la población ayudará a aclarar este último punto. Esta relación se ha observado en el contexto de las campañas de vacunación, durante las cuales los picos anuales de los brotes virales cambian a finales de año a medida que se inmuniza a la población [57]. Por esta razón, es probable que la frecuencia de los brotes a lo largo de los años afecte el momento de los brotes individuales y puede hacer que los brotes de arbovirus se sincronicen.

Igualmente, importante es la identificación de virus que podrían extenderse a través de fronteras internacionales. En nuestro estudio, observamos un vínculo epidemiológico entre los brotes de Zika en Haití y la región suroeste

de la República Dominicana. Es probable que esta conexión se extienda a otros virus transmitidos por mosquitos, como el virus Mayaro y el virus del Nilo Occidental, que se han informado en Haití [58–60]. El primero puede ser transmitido por mosquitos *Aedes* [12,61–63], y el segundo ha sido aislado de reservorios zoonóticos en República Dominicana [64]. De manera similar, los informes de viajeros internacionales que transportan virus transmitidos por mosquitos desde la República Dominicana a sus países de origen demuestran que las brechas en la vigilancia tienen implicaciones globales [65–70]. Por lo tanto, comprender los patrones de transmisión de los virus y desarrollar un plan internacional unificado para combatirlos antes de que causen un brote ayudará a mitigar el potencial de tal evento.

Nuestros hallazgos sugieren que los casos de dengue no se notificaron después del brote de Zika en 2016; sin embargo, hay una serie de posibles explicaciones para la ostensible disminución de los casos de dengue que deberían explorarse. Las medidas generalizadas de control de mosquitos motivadas por el brote de Zika podrían haber limitado la propagación del dengue más tarde ese año. Aunque es plausible, esta línea de razonamiento no explica por qué se observó una disminución similar posterior al Zika en otros países de las Américas [71], ni por qué hubo un resurgimiento de casos de dengue en 2019. Una segunda explicación es que las infecciones por Zika confieren algún nivel de inmunidad temporal a las posteriores infecciones por dengue [45,72,73]. Esta teoría no puede explicar la pequeña cantidad de casos de dengue notificados en 2014 después del brote de chikungunya, ya que el agente etiológico de esa enfermedad es un alfavirus, y asume tasas de ataque muy altas y una subnotificación extensa de Zika en 2016 para haber logrado suficientes niveles de inmunidad colectiva. En cambio, nuestros hallazgos sugieren que la infección previa por Zika protege contra las infecciones sintomáticas del dengue porque observamos una correlación positiva significativa entre las tasas de ataque de dengue dentro de las provincias a través de los brotes. Dado que la inmunidad de reacción cruzada entre los serotipos del dengue está bien documentada [74,75], nuestros datos sugieren que una relación similar entre el dengue y el Zika no daría lugar a una disminución generalizada de los casos. Más bien, la transmisión del dengue podría haber persistido razonablemente sin ser detectada si la mayoría de los infectados no hubieran sido hospitalizados. De ser cierta, esta hipótesis explicaría por qué los picos estacionales en los casos notificados de enfermedad febril persistieron en 2017 y 2018, y por qué los CFR entre los casos notificados de dengue parecían estar elevados en las Temporadas 5 y 6 si el número real de casos no se informó. Para comprender mejor estas complejas interacciones, la recopilación de información sobre el serotipo debe incorporarse a los esfuerzos actuales de vigilancia del dengue.

Hay algunas limitaciones importantes en nuestro estudio. En primer lugar, nuestro conjunto de datos incluía datos de casos de chikungunya y Zika de la ola inicial de cada enfermedad y, por lo tanto, no podemos comparar la dinámica temporal y espacial de estas enfermedades entre estaciones. Después de estos brotes iniciales, las pruebas de diagnóstico para estas enfermedades han cesado en gran medida. Si bien el número de casos de estas enfermedades notificados en la República Dominicana se ha reducido a cero, se desconoce la verdadera carga de morbilidad. Los estudios futuros deben investigar si estos virus han seguido circulando sin ser detectados en el país y si su dinámica espacio-temporal se ha sincronizado desde entonces con la del virus del dengue. En segundo lugar, el sistema de notificación de casos sospechosos de chikungunya difería del utilizado para casos sospechosos de Zika. Durante el brote de chikungunya, la mayoría de los casos de enfermedades febriles sin causa aparente se clasificaron inicialmente como casos sospechosos de chikungunya. Por esta razón, el número de casos reportados por la Organización Panamericana de la Salud (OPS) y el Ministerio de Salud fue significativamente mayor que los que aquí reportamos [76]. Nuestros datos de casos de chikungunya contienen un número desproporcionado de niños en el grupo de edad <1 año, lo que indica que la difusión de las pruebas de diagnóstico puede haberse sesgado hacia grupos de alto riesgo. En tercer lugar, nuestros hallazgos demuestran que existió una relación epidemiológica entre la República Dominicana y Haití durante la epidemia de Zika en 2016, pero no podemos determinar la direccionalidad del movimiento transfronterizo del virus sin datos genómicos del virus. Sin embargo, dado que los mosquitos no reconocen fronteras políticas, es posible que los propios vectores infectados se muevan entre países. Es más probable que el movimiento humano entre los dos países facilitado por la carretera principal (Fig. 1e) impulse la propagación internacional de los virus a mayor distancia [77]. Independientemente del

mecanismo exacto, se puede suponer que en el futuro se producirá una propagación bidireccional de enfermedades transmitidas por mosquitos a menos que se implementen las medidas de control y vigilancia binacionales adecuadas. Por último, nuestro análisis se centró en la transmisión del virus por los mosquitos *Aedes aegypti,* pero es posible que otros mosquitos vectores contribuyan a la propagación de los brotes que investigamos. Específicamente, *Aedes albopictus* puede haber jugado un papel clave en la transmisión de chikungunya [78,79]. Se dispone de datos limitados sobre la distribución de mosquitos vectores relevantes en el país [80,81] y se necesita una vigilancia entomológica más amplia para abordar mejor esta cuestión.

En conjunto, nuestro estudio demuestra que la vigilancia de las enfermedades transmitidas por mosquitos debe mantenerse durante los períodos en los que la transmisión parece ser baja porque los patrones en los casos de dengue notificados son malos indicadores de la dinámica futura de los brotes emergentes de virus transmitidos por mosquitos. Los síntomas notificados y los datos demográficos de los casos pueden ser útiles para identificar cambios en la prevalencia de la enfermedad, pero muchas características clínicas, especialmente la fiebre, que hemos observado son probablemente una función de los algoritmos de notificación y diagnóstico utilizados durante un brote. Se necesitan informes activos de nuevos casos de dengue, chikungunya y Zika y el despliegue más amplio de diagnósticos para enfermedades emergentes para determinar perfiles de casos más precisos. Los brotes de enfermedades tropicales emergentes son una amenaza para la salud pública del Caribe, y las enfermedades endémicas como el dengue precipitan crisis de salud con una frecuencia cada vez mayor. Dada la omnipresencia de las enfermedades transmitidas por mosquitos en los climas tropicales, se deben implementar sistemas de vigilancia sostenibles en lugar de medidas reaccionarias de control de enfermedades para prevenir crisis futuras.

## MÉTODOS

### Descripción de datos

Los datos de los casos sospechosos y confirmados de dengue, chikungunya y Zika notificados entre 2012-2018 se extrajeron de los Informes semanales del Ministerio de Salud de la República Dominicana (digepi.gob.do). Se solicitaron características demográficas y clínicas despersonalizadas de los casos a la Dirección Nacional de Estadística del Ministerio de Salud (MoH) (saip.gob.do). Los datos se organizaron por fecha de inicio del informe de síntomas.

La Dirección Nacional de Epidemiología del Ministerio de Salud recopila informes de casos de dengue mediante vigilancia pasiva de manera semanal en su formulario Epi 1 dentro de su Plataforma Digital del Sistema Nacional de Vigilancia Epidemiológica (SINAVE) para todos los entornos de atención médica en todo el país. Estos datos se recopilaron en informes semanales enviados a su sitio web (digepi.gob.do) y organizados por provincia y semana de casos notificados. Los datos recopilados por el Ministerio de Salud incluyen edad, sexo, provincia y municipio de residencia, fecha de inicio de los síntomas, resultado clínico y sintomatología. El Ministerio de Salud incluyó informes obligatorios de la infección por chikungunya presunta y confirmada hasta 2014 y el virus del Zika durante 2016, sin seguir haciéndolo a partir de entonces.

Se solicitaron datos semanales sobre enfermedades febriles agudas a nivel provincial y municipal de 2012-2018 a la Dirección Nacional de Epidemiología del Ministerio de Salud (saip.gob.do), que recopila los datos como parte de un sistema de vigilancia pasiva. En 2014, el Ministerio de Salud incrementó sus esfuerzos para identificar casos de enfermedad febril [46].

Debido a que los datos descritos anteriormente están disponibles públicamente por solicitud a las instituciones correspondientes y consisten en datos despersonalizados, no tuvimos que buscar la aprobación ética de nuestro Comité de Ética Institucional (CEI).

Las variables climatológicas diarias de temperatura media y humedad relativa para 5 ciudades (Santo Domingo, Distrito Nacional; Barahona, Barahona; La Unión, Puerto Plata; Cabrera, María Trinidad Sánchez; Santiago, Santiago) se recolectaron de la base de datos de la Oficina Nacional de Meteorología (ONAMET) (saip.gob.do) desde enero de 2012 hasta diciembre de 2018. Los datos climatológicos horarios para Bonao, Monseñor Nouel para el mismo período de tiempo se obtuvieron de openweathermap.org. Los datos de población se extrajeron del Noveno Censo Nacional de Población y Hogares. Este censo fue realizado en 2010 por la Oficina Nacional de Estadísticas [82].

Los casos notificados de dengue, chikungunya y zika para Haití, Bolivia, Jamaica y Venezuela se extrajeron del sitio web de la Organización Panamericana de la Salud y de la base de datos PLISA [83]. Los casos de dengue y zika, que se notificaron por semana epidemiológica, se agregaron por mes para permitir una comparación directa con los casos de chikungunya, que se informaron por mes.

**Tiempo para el pico del brote**

Utilizando los datos informados semanalmente recopilados por el Ministerio de Salud, calculamos el tiempo máximo para los brotes provinciales identificando primero la semana epidemiológica en la que se notificó el primer caso del brote nacional (chikungunya: SE 6, Zika: SE 1, dengue (3 ): SE 14) y luego contando el número de semanas transcurridas hasta que cada una de las 32 provincias notificó el número máximo de casos para el brote correspondiente. El tiempo medio hasta el pico de cada brote se comparó mediante una prueba T no aparejada implementada en Prism v8.4.2.

**Potencial de transmisión**

Calculamos el potencial de transmisión semanal (índice P) con el enfoque bayesiano desarrollado por Obolski et al. [19]. Brevemente, extrajimos la temperatura promedio diaria y la humedad relativa de 6 ciudades de la base de datos de la Oficina Nacional de Meteorología o, en el caso de la provincia de Monseñor Nouel, openweathermap.org. En raras ocasiones, la temperatura o la humedad no estaban disponibles para un día determinado. En estos casos, promediamos la variable respectiva desde la misma fecha en los 6 años restantes. Hicimos esto durante 14 días para Barahona y Puerto Plata, 3 días para Santiago y 1 día para María Trinidad Sánchez.

Luego usamos el paquete R MVSE y los antecedentes entomológicos y epidemiológicos documentados en la literatura (Tabla Suplementaria 1) para calcular el potencial de transmisión diario, a partir del cual calculamos las medias semanales para la duración de nuestro período de estudio [19]. Descubrimos que el modelo era razonablemente robusto para una variedad de valores previos para cada uno de los parámetros y, por lo tanto, elegimos utilizar estimaciones breves del período infeccioso y de incubación humana para informar el modelo. Adoptamos nuestra estimación previa de la esperanza de vida humana en la República Dominicana basada en estimaciones del Programa de las Naciones Unidas para el Desarrollo [84], pero por lo demás usamos los mismos antecedentes que informaron Oboloski et al. (Tabla Suplementaria 1) [19].

**Estimaciones del número de reproducción efectivo ($R_{eff}$)**

Estimamos $R_{eff}$ para los cinco brotes utilizando el método desarrollado por Lipsitch et al. [44] para ajustar un modelo de regresión lineal a los datos de nuestro caso (Eq. 1).

$$R_{eff} = r^2 * (1 - f) * f * v^2 + r * v + 1 \quad \text{Eq. 1}$$

Usamos un rango de valores para períodos latentes e infecciosos que fueron bien informados en la literatura para calcular f, la proporción del período de incubación en el intervalo de serie, yv, el intervalo de serie medido en semanas (Tabla Suplementaria 2). Específicamente, estimamos los valores mínimos de $R_{eff}$ utilizando las estimaciones mínimas del período de incubación y el intervalo de serie para cada enfermedad (Tabla Suplementaria 2), para calcular f y v, respectivamente. Repetimos este proceso con las estimaciones máximas

para cada intervalo para obtener los valores máximos de $R_{eff}$. La mediana de cada par de valores se indica mediante barras horizontales en la Fig. 3a. Obtuvimos r, la tasa de crecimiento epidémico, ajustando un modelo lineal generalizado a los casos acumulados reportados para cada brote y extrayendo la pendiente del modelo. Todos los cálculos se realizaron utilizando R v4.0.0.

**Tasas de ataque ajustadas y regresión lineal**

Las tasas de ataque a nivel provincial por brote se ajustaron por edad y sexo mediante el método de estandarización directa, con los casos notificados diariamente como entrada y la población nacional como población de referencia.

Calculamos el coeficiente de correlación R de Pearson para las tasas de ataque a nivel de provincia entre varios pares de brotes (Fig. 4a, b). Un análisis de valores atípicos mostró que la eliminación de los valores atípicos no afectó sustancialmente el tamaño o la importancia de los coeficientes de correlación, por lo que incluimos todos los puntos de datos.

**Puntaje de idoneidad de *Aedes aegypti* (AaS)**

El AaS se calculó para cada mes utilizando datos climáticos y de uso de la tierra en una escala de 5 km x 5 km recopilados de 1970 y 2000 y compilados por WorldClim como lo describen Kraemer et al. [85]. Se extrajeron los puntajes de idoneidad mensuales y se promediaron por provincia en Rv4.0.0.

**AGRADECIMIENTOS**

Agradecemos a C. Vogels, A. Brito, J. Fauver, C. Kalinich, I. Ott, S. Lapidus, K. Gangavarapu, J. Pack y S. Taylor por sus comentarios y / o ayuda con la metodología. El estudio fue financiado en parte por el premio Hecht Global Health Faculty Network Award otorgado a N.D.G. del Instituto de Salud Global de Yale.

**CONTRIBUCIONES DE AUTOR**

M.E.P., L.T. y N.D.G. concebido del estudio. M.E.P. y R. E. diseñaron los análisis. M.E.P., R.E., J.L., M.U.G.K., R.P., N.D.G. y L.T. todos contribuyeron al análisis de datos y a la redacción del artículo.

**CONFLICTO DE INTERESES**

Los autores declaran no tener conflictos de intereses.

**MATERIALES Y CORRESPONDENCIA**

M.E.P (mary.petrone@yale.edu) y L.T. (l.tapia@prof.unibe.edu.do) son autores correspondientes.

**DISPONIBILIDAD DE DATOS**

Con la excepción de los datos globales de vigilancia y AaS, los datos fuente de este manuscrito están disponibles en GitHub (https://github.com/grubaughlab/Paper_arbovirus_Epi_DR). Los datos de vigilancia están disponibles a solicitud del Ministerio de Salud de República Dominicana. Los valores globales de AaS pueden estar disponibles a pedido.

**DISPONIBILIDAD DE CÓDIGOS**

Los scripts de R de este manuscrito están disponibles en GitHub (https://github.com/grubaughlab/Paper_arbovirus_Epi_DR).

## FIGURAS SUPLEMENTARIAS

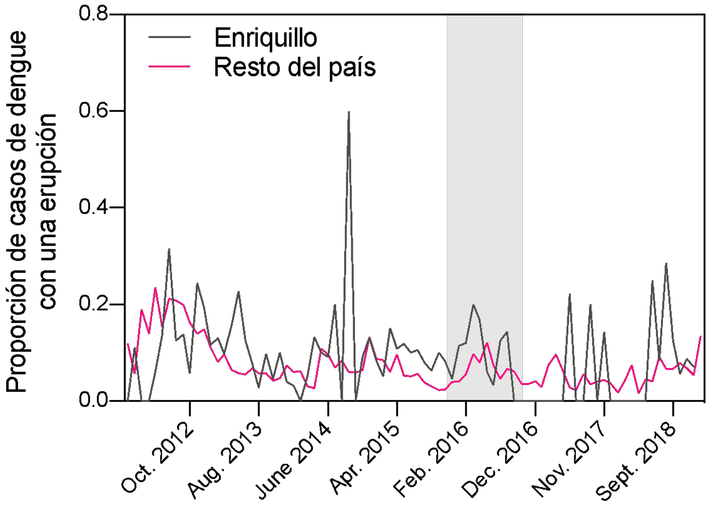


**Figura Suplementaria 1: La proporción de casos de dengue que informaron haber experimentado una erupción alcanzó su punto máximo antes en la región suroeste de Enriquillo en comparación con el resto del país.** Se sumó el número de casos de dengue con exantema para cada mes. El cuadro gris indica cuándo ocurrió el brote de Zika.

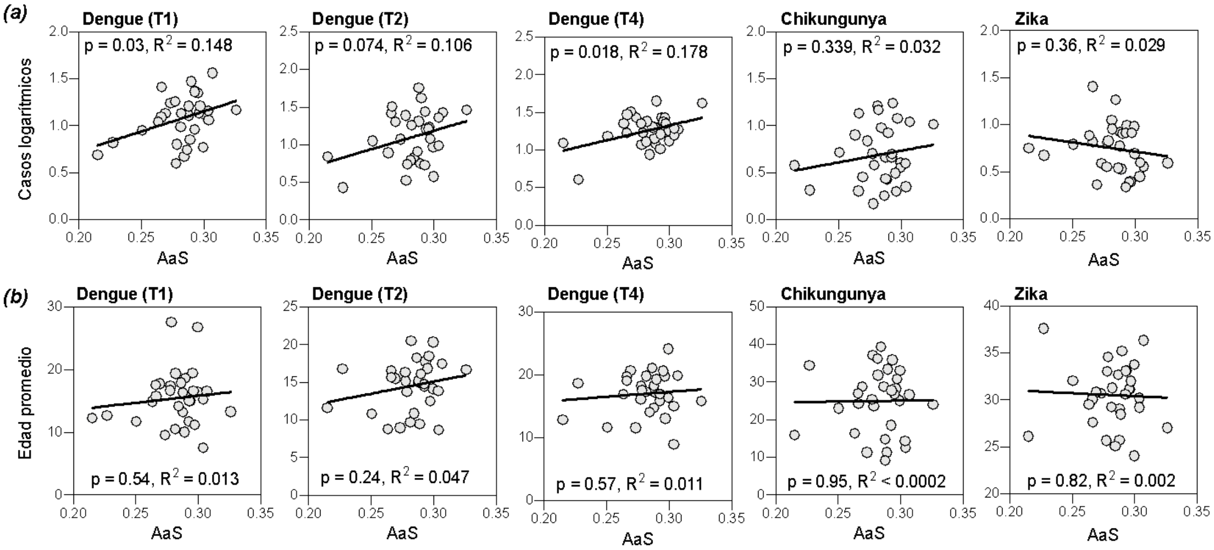


**Figura Suplementaria 2: Correlación mínima entre las tasas de ataque logarítmicas ajustadas, la fuerza de la infección y la abundancia promedio de mosquitos (AaS) por provincia.** En ambos análisis, se excluyó una provincia, Elías Piña, debido a la supuesta subnotificación sustancial. Esta provincia, que tiene una población de 63,250 según el censo de 2010, reportó 1 caso de dengue durante la temporada 1 y 1 caso de chikungunya durante ese brote. (a) Tasas de ataque con ajuste logarítmico frente a AaS (b) Edad media de infección frente a AaS (fuerza de la infección = edad media de la infección-1)

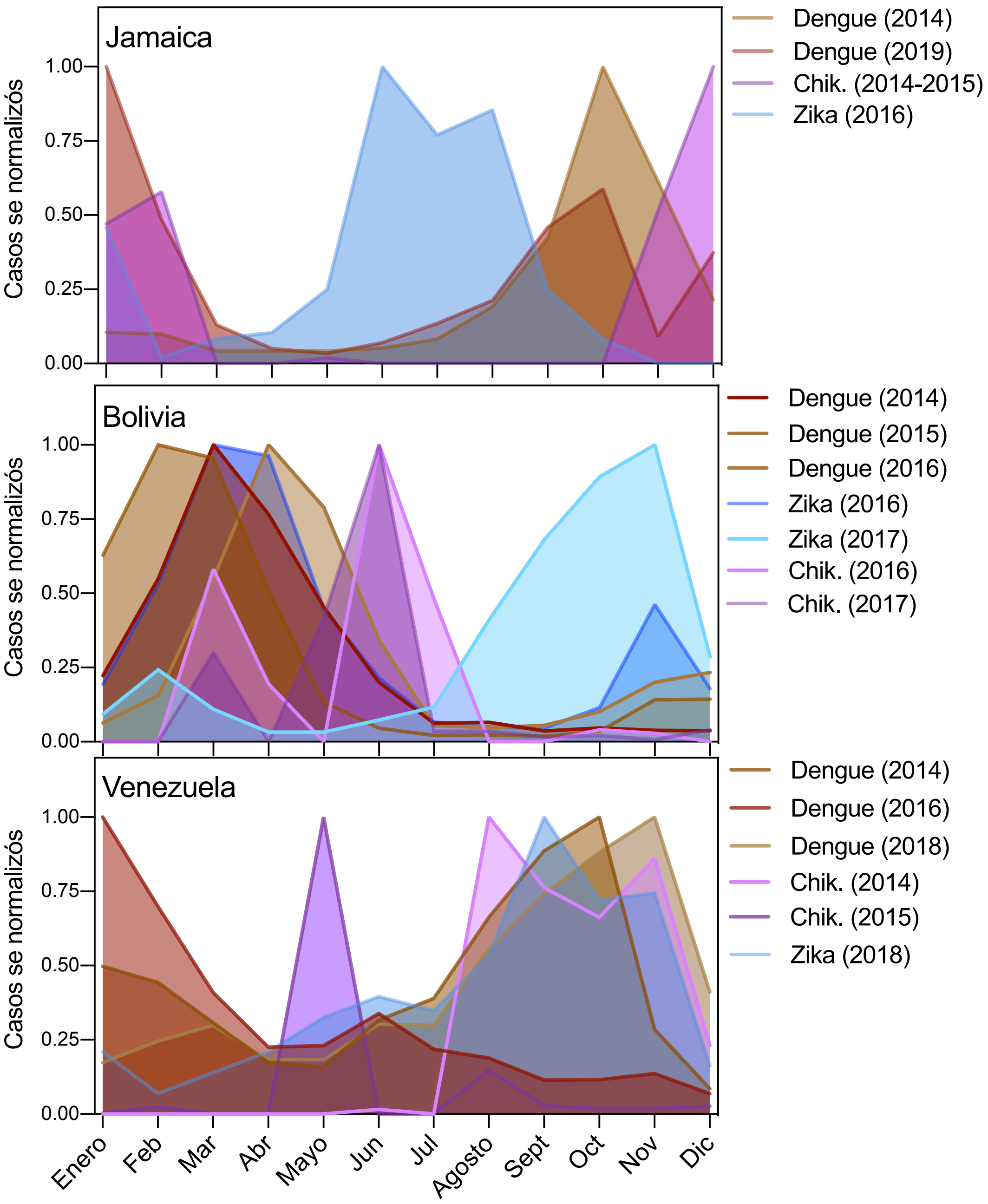


**Figura Suplementaria 3: Asincronicidad de los brotes notificados de dengue, chikungunya y Zika en tres países de las Américas. Los casos se normalizaron por brote individual.** Se obtuvieron datos de casos mensuales (chikungunya) y semanales (dengue y Zika) del sitio web de la Organización Panamericana de la Salud (https://www.paho.org/en).

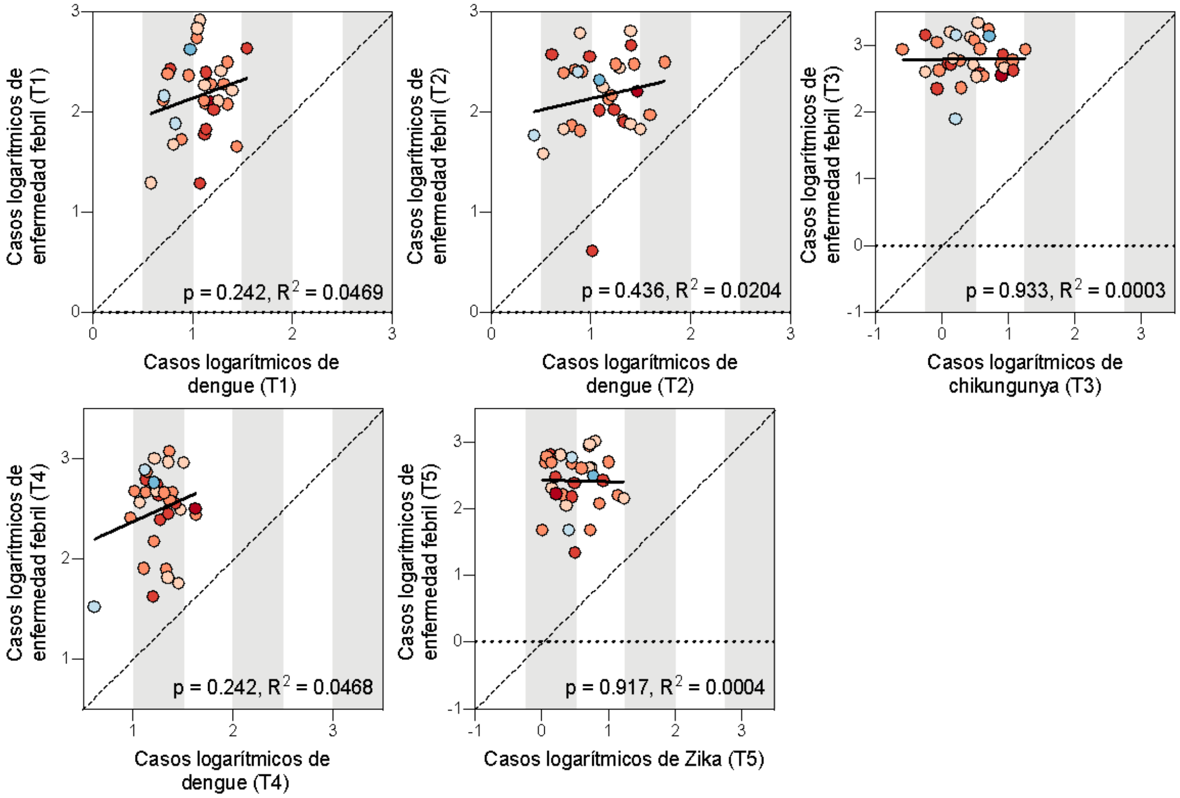


**Figura Suplementaria 4: No hay una correlación significativa entre la enfermedad y las tasas de ataque de enfermedad febril informadas por provincia durante los brotes de dengue, chikungunya y Zika.** Se calcularon las tasas de ataque logarítmicas ajustadas a la población por cada 10,000 personas para cada una de las 31 provincias utilizando los datos de enfermedades y enfermedades febriles informados durante cada brote. Cada punto de datos representa una provincia. Por las razones expuestas anteriormente, la provincia Elías Piña fue excluida del análisis. Los colores corresponden al esquema utilizado en la Fig. 4c.

## Tabla Suplementaria 1. A priori para estimaciones del Índice P

| **Parámetro** | **Distribución *a priori*** | **Fuentes** |
|---|---|---|
| Periodo de Incubación Humano | Gaussian (media = 5, SD = 1) | 23,86,87 |
| Periodo Infeccioso Humano | Gaussian (media = 5, SD = 1) | 23,86,87 |
| Expectativa de Vida Humana | Gaussian (media = 73, SD = 2) | 84 |
| Probabilidad de Transmisión (humano a mosquito) | Gaussian (media = 0.5, SD = 0.01) | 19 |
| Tasa de Picaduras de Mosquito | Gaussian (media = 0.25, SD = 0.05) | 88,89 |
| Periodo de Incubación Extrínseca | Gaussian (media = 7, SD = 2) | 86,90,91 |
| Expectativa de Vida de Mosquito | Gaussian (media = 14, SD = 3) | 92,93 |

**Tabla Suplementaria 2. Parámetros de estimación de $R_{eff}$.**

| | Periodo de Incubación (días) | Periodo Infeccioso (días) | Intervalo de Series (días) | Fuentes |
|---|---|---|---|---|
| Dengue | 4-6 | 9-11 | 13-17 | 94 |
| Zika | 3-14 | 7-8 | 10-23 | 95,96 |
| Chikungunya | 2-12 | 7-9 | 9-21 | 97,98 |

## REFERENCIAS


1. Cao-Lormeau, V.-M. Tropical Islands as New Hubs for Emerging Arboviruses. *Emerg. Infect. Dis.* **22**, 913–915 (2016).
2. Mavian, C. *et al.* Islands as Hotspots for Emerging Mosquito-Borne Viruses: A One-Health Perspective. *Viruses* **11**, (2018).
3. Musso, D., Rodriguez-Morales, A. J., Levi, J. E., Cao-Lormeau, V.-M. & Gubler, D. J. Unexpected outbreaks of arbovirus infections: lessons learned from the Pacific and tropical America. *Lancet Infect. Dis.* **18**, e355–e361 (2018).
4. Cassadou, S. *et al.* Emergence of chikungunya fever on the French side of Saint Martin island, October to December 2013. *Euro Surveill.* **19**, (2014).
5. Dorléans, F. *et al.* Outbreak of Chikungunya in the French Caribbean Islands of Martinique and Guadeloupe: Findings from a Hospital-Based Surveillance System (2013–2015). *Am. J. Trop. Med. Hyg.* **98**, 1819–1825 (2018).
6. Halstead, S. B. Reappearance of chikungunya, formerly called dengue, in the Americas. *Emerg. Infect. Dis.* **21**, 557–561 (2015).
7. Faria, N. R. *et al.* Zika virus in the Americas: Early epidemiological and genetic findings. *Science* **352**, 345–349 (2016).
8. Grubaugh, N. D., Faria, N. R., Andersen, K. G. & Pybus, O. G. Genomic Insights into Zika Virus Emergence and Spread. *Cell* **172**, 1160–1162 (2018).
9. Metsky, H. C. *et al.* Zika virus evolution and spread in the Americas. *Nature* **546**, 411–415 (2017).
10. Ganjian, N. & Riviere-Cinnamond, A. Mayaro virus in Latin America and the Caribbean. *Rev. Panam. Salud Publica* **44**, e14 (2020).
11. Hotez, P. J. & Murray, K. O. Dengue, West Nile virus, chikungunya, Zika-and now Mayaro? *PLoS Negl. Trop. Dis.* **11**, e0005462 (2017).
12. Long, K. C. *et al.* Experimental transmission of Mayaro virus by Aedes aegypti. *Am. J. Trop. Med. Hyg.* **85**, 750–757 (2011).
13. Lorenz, C., Freitas Ribeiro, A. & Chiaravalloti-Neto, F. Mayaro virus distribution in South America. *Acta Trop.* **198**, 105093 (2019).
14. Weaver, S. C. & Reisen, W. K. Present and future arboviral threats. *Antiviral Res.* **85**, 328–345 (2010).
15. *Suspected dengue cases by epidemiological week for countries and territories of the America*. https://www.paho.org/data/index.php/en/mnu-topics/indicadores-dengue-en/dengue-nacional-en/252-dengue-pais-ano-en.html?start=2.
16. *Five-fold increase in dengue cases in the Americas over the past decade*. https://www.paho.org/hq/index.php?option=com_content&view=article&id=9657:2014-los-casos-dengue-americas- (2014).
17. Kraemer, M. U. G. *et al.* Past and future spread of the arbovirus vectors Aedes aegypti and Aedes albopictus. *Nat Microbiol* **4**, 854–863 (2019).
18. Kraemer, M. U. G. *et al.* The global distribution of the arbovirus vectors Aedes aegypti and Ae. albopictus. *Elife* **4**, e08347 (2015).
19. Obolski, U. *et al.* MVSE : An R-package that estimates a climate-driven mosquito-borne viral suitability index. *Methods Ecol. Evol.* **10**, 1357–1370 (2019).
20. Hamlet, A. *et al.* The seasonal influence of climate and environment on yellow fever transmission across Africa. *PLoS Negl. Trop. Dis.* **12**, e0006284 (2018).
21. Do, T. T. T., Martens, P., Luu, N. H., Wright, P. & Choisy, M. Climatic-driven seasonality of emerging dengue fever in Hanoi, Vietnam. *BMC Public Health* **14**, 1078 (2014).
22. Perez-Guzman, P. N. *et al.* Measuring Mosquito-borne Viral Suitability in Myanmar and Implications for Local Zika Virus Transmission. *PLoS Curr.* **10**, (2018).

23. Lourenço, J. *et al.* Epidemiological and ecological determinants of Zika virus transmission in an urban setting. *eLife* vol. 6 (2017).

24. *Dengue serotypes by year for countries and territories of the Americas*. https://www.paho.org/data/index.php/es/temas/indicadores-dengue/dengue-nacional/549-dengue-serotypes-es.html.

25. Bowman, L. R., Rocklöv, J., Kroeger, A., Olliaro, P. & Skewes, R. A comparison of Zika and dengue outbreaks using national surveillance data in the Dominican Republic. *PLoS Negl. Trop. Dis.* **12**, e0006876 (2018).

26. Lindsey, N. P., Staples, J. E. & Fischer, M. Chikungunya Virus Disease among Travelers-United States, 2014-2016. *Am. J. Trop. Med. Hyg.* **98**, 192–197 (2018).

27. Zingman, M. A., Paulino, A. T. & Payano, M. P. Clinical manifestations of chikungunya among university professors and staff in Santo Domingo, the Dominican Republic. *Rev. Panam. Salud Publica* **41**, e64 (2017).

28. Duffy, M. R. *et al.* Zika virus outbreak on Yap Island, Federated States of Micronesia. *N. Engl. J. Med.* **360**, 2536–2543 (2009).

29. He, A., Brasil, P., Siqueira, A. M., Calvet, G. A. & Kwatra, S. G. The Emerging Zika Virus Threat: A Guide for Dermatologists. *Am. J. Clin. Dermatol.* **18**, 231–236 (2017).

30. Langsjoen, R. M. *et al.* Molecular Virologic and Clinical Characteristics of a Chikungunya Fever Outbreak in La Romana, Dominican Republic, 2014. *PLoS Negl. Trop. Dis.* **10**, e0005189 (2016).

31. Martinez, J. D., Garza, J. A. C. la & Cuellar-Barboza, A. Going Viral 2019: Zika, Chikungunya, and Dengue. *Dermatol. Clin.* **37**, 95–105 (2019).

32. Pineda, C., Muñoz-Louis, R., Caballero-Uribe, C. V. & Viasus, D. Chikungunya in the region of the Americas. A challenge for rheumatologists and health care systems. *Clin. Rheumatol.* **35**, 2381–2385 (2016).

33. Rosario, V. *et al.* Chikungunya infection in the general population and in patients with rheumatoid arthritis on biological therapy. *Clin. Rheumatol.* **34**, 1285–1287 (2015).

34. Grubaugh, N. D. *et al.* Tracking virus outbreaks in the twenty-first century. *Nat Microbiol* **4**, 10–19 (2019).

35. Rodrigues Faria, N. *et al.* Epidemiology of Chikungunya Virus in Bahia, Brazil, 2014-2015. *PLoS Curr.* **8**, (2016).

36. Liu-Helmersson, J., Stenlund, H., Wilder-Smith, A. & Rocklöv, J. Vectorial capacity of Aedes aegypti: effects of temperature and implications for global dengue epidemic potential. *PLoS One* **9**, e89783 (2014).

37. Chan, M. & Johansson, M. A. The incubation periods of Dengue viruses. *PLoS One* **7**, e50972 (2012).

38. Mordecai, E. A. *et al.* Detecting the impact of temperature on transmission of Zika, dengue, and chikungunya using mechanistic models. *PLoS Negl. Trop. Dis.* **11**, e0005568 (2017).

39. Winokur, O. C., Main, B. J., Nicholson, J. & Barker, C. M. Impact of temperature on the extrinsic incubation period of Zika virus in Aedes aegypti. *PLoS Negl. Trop. Dis.* **14**, e0008047 (2020).

40. Faria, N. R. *et al.* Establishment and cryptic transmission of Zika virus in Brazil and the Americas. *Nature* **546**, 406–410 (2017).

41. Cauchemez, S. *et al.* Local and regional spread of chikungunya fever in the Americas. *Euro Surveill.* **19**, 20854 (2014).

42. Nishiura, H., Kinoshita, R., Mizumoto, K., Yasuda, Y. & Nah, K. Transmission potential of Zika virus infection in the South Pacific. *Int. J. Infect. Dis.* **45**, 95–97 (2016).

43. Liu, Y. *et al.* Reviewing estimates of the basic reproduction number for dengue, Zika and chikungunya across global climate zones. *Environ.*

*Res.* **182**, 109114 (2020).

44. Lipsitch, M. *et al.* Transmission dynamics and control of severe acute respiratory syndrome. *Science* **300**, 1966–1970 (2003).
45. Rodriguez-Barraquer, I. *et al.* Impact of preexisting dengue immunity on Zika virus emergence in a dengue endemic region. *Science* **363**, 607–610 (2019).
46. *Plan de preparación y respuesta frente a brotes de Fiebre Chikungunya*. (2014).
47. Low, G. K.-K., Ogston, S. A., Yong, M.-H., Gan, S.-C. & Chee, H.-Y. Global dengue death before and after the new World Health Organization 2009 case classification: A systematic review and meta-regression analysis. *Acta Trop.* **182**, 237–245 (2018).
48. Freitas, A. R. R., Alarcón-Elbal, P. M., Paulino-Ramírez, R. & Donalisio, M. R. Excess mortality profile during the Asian genotype chikungunya epidemic in the Dominican Republic, 2014. *Trans. R. Soc. Trop. Med. Hyg.* **112**, 443–449 (2018).
49. Imai, N., Dorigatti, I., Cauchemez, S. & Ferguson, N. M. Estimating dengue transmission intensity from sero-prevalence surveys in multiple countries. *PLoS Negl. Trop. Dis.* **9**, e0003719 (2015).
50. Fauver, J. R. *et al.* The Use of Xenosurveillance to Detect Human Bacteria, Parasites, and Viruses in Mosquito Bloodmeals. *Am. J. Trop. Med. Hyg.* **97**, 324–329 (2017).
51. Fauver, J. R. *et al.* Xenosurveillance reflects traditional sampling techniques for the identification of human pathogens: A comparative study in West Africa. *PLoS Negl. Trop. Dis.* **12**, e0006348 (2018).
52. Grubaugh, N. D. *et al.* Xenosurveillance: a novel mosquito-based approach for examining the human-pathogen landscape. *PLoS Negl. Trop. Dis.* **9**, e0003628 (2015).
53. Grubaugh, N. D. *et al.* Travel Surveillance and Genomics Uncover a Hidden Zika Outbreak during the Waning Epidemic. *Cell* **178**, 1057–1071.e11 (2019).
54. Vogels, C. B. F. *et al.* Arbovirus coinfection and co-transmission: A neglected public health concern? *PLoS Biol.* **17**, e3000130 (2019).
55. Bisanzio, D. *et al.* Spatio-temporal coherence of dengue, chikungunya and Zika outbreaks in Merida, Mexico. *PLoS Negl. Trop. Dis.* **12**, e0006298 (2018).
56. Freitas, L. P., Cruz, O. G., Lowe, R. & Sá Carvalho, M. Space–time dynamics of a triple epidemic: dengue, chikungunya and Zika clusters in the city of Rio de Janeiro. *Proceedings of the Royal Society B: Biological Sciences* **286**, 20191867 (2019).
57. Shioda, K. *et al.* Identifying signatures of the impact of rotavirus vaccines on hospitalizations using sentinel surveillance data from Latin American countries. *Vaccine* **38**, 323–329 (2020).
58. Blohm, G. *et al.* Mayaro as a Caribbean traveler: Evidence for multiple introductions and transmission of the virus into Haiti. *Int. J. Infect. Dis.* **87**, 151–153 (2019).
59. Lednicky, J. *et al.* Mayaro Virus in Child with Acute Febrile Illness, Haiti, 2015. *Emerg. Infect. Dis.* **22**, 2000–2002 (2016).
60. Weppelmann, T. A. *et al.* A Tale of Two Flaviviruses: A Seroepidemiological Study of Dengue Virus and West Nile Virus Transmission in the Ouest and Sud-Est Departments of Haiti. *Am. J. Trop. Med. Hyg.* **96**, 135–140 (2017).
61. Kantor, A. M., Lin, J., Wang, A., Thompson, D. C. & Franz, A. W. E. Infection Pattern of Mayaro Virus in Aedes aegypti (Diptera: Culicidae) and Transmission Potential of the Virus in Mixed Infections With Chikungunya Virus. *J. Med. Entomol.* **56**, 832–843 (2019).
62. Pereira, T. N., Carvalho, F. D., De Mendonça, S. F., Rocha, M. N. & Moreira, L. A. Vector competence of Aedes aegypti, Aedes albopictus, and

Culex quinquefasciatus mosquitoes for Mayaro virus. *PLoS Negl. Trop. Dis.* **14**, e0007518 (2020).

63. Wiggins, K., Eastmond, B. & Alto, B. W. Transmission potential of Mayaro virus in Florida Aedes aegypti and Aedes albopictus mosquitoes. *Med. Vet. Entomol.* **32**, 436–442 (2018).
64. Komar, O. *et al.* West Nile Virus Survey of Birds and Mosquitoes in the Dominican Republic. *Vector-Borne and Zoonotic Diseases* vol. 5 120–126 (2005).
65. Barzon, L. *et al.* Isolation of infectious Zika virus from saliva and prolonged viral RNA shedding in a traveller returning from the Dominican Republic to Italy, January 2016. *Euro Surveill.* **21**, 30159 (2016).
66. Díaz-Menéndez, M. *et al.* Initial experience with imported Zika virus infection in Spain. *Enfermedades Infecciosas y Microbiología Clínica* vol. 36 4–8 (2018).
67. Duijster, J. W. *et al.* Zika virus infection in 18 travellers returning from Surinam and the Dominican Republic, The Netherlands, November 2015–March 2016. *Infection* **44**, 797–802 (2016).
68. Goncé, A. *et al.* Spontaneous Abortion Associated with Zika Virus Infection and Persistent Viremia. *Emerg. Infect. Dis.* **24**, 933–935 (2018).
69. Millman, A. J. *et al.* Chikungunya and Dengue Virus Infections Among United States Community Service Volunteers Returning from the Dominican Republic, 2014. *Am. J. Trop. Med. Hyg.* **94**, 1336–1341 (2016).
70. Requena-Méndez, A. *et al.* Cases of chikungunya virus infection in travellers returning to Spain from Haiti or Dominican Republic, April-June 2014. *Euro Surveill.* **19**, 20853 (2014).
71. Perez, F. *et al.* The decline of dengue in the Americas in 2017: discussion of multiple hypotheses. *Tropical Medicine & International Health* vol. 24 442–453 (2019).
72. Gordon, A. *et al.* Prior dengue virus infection and risk of Zika: A pediatric cohort in Nicaragua. *PLoS Med.* **16**, e1002726 (2019).
73. Ribeiro, G. S. *et al.* Does immunity after Zika virus infection cross-protect against dengue? *Lancet Glob Health* **6**, e140–e141 (2018).
74. Katzelnick, L. C., Montoya, M., Gresh, L., Balmaseda, A. & Harris, E. Neutralizing antibody titers against dengue virus correlate with protection from symptomatic infection in a longitudinal cohort. *Proc. Natl. Acad. Sci. U. S. A.* **113**, 728–733 (2016).
75. Tsang, T. K. *et al.* Effects of infection history on dengue virus infection and pathogenicity. *Nat. Commun.* **10**, 1246 (2019).
76. *República Dominicana Chikungunya*. https://www.paho.org/dor/images/stories/archivos/chikungunya/boletin_chikv_no-13_2014_8_20.pdf?ua=1 (2014).
77. Verdonschot, P. F. M. & Besse-Lototskaya, A. A. Flight distance of mosquitoes (Culicidae): A metadata analysis to support the management of barrier zones around rewetted and newly constructed wetlands. *Limnologica* **45**, 69–79 (2014).
78. Lamballerie, X. de *et al.* Chikungunya virus adapts to tiger mosquito via evolutionary convergence: a sign of things to come? *Virology Journal* vol. 5 33 (2008).
79. Vazeille, M. *et al.* Two Chikungunya isolates from the outbreak of La Reunion (Indian Ocean) exhibit different patterns of infection in the mosquito, Aedes albopictus. *PLoS One* **2**, e1168 (2007).
80. González, M. A. *et al.* Micro-environmental features associated to container-dwelling mosquitoes (Diptera: Culicidae) in an urban cemetery of the Dominican Republic. *Revista de Biología Tropical* vol. 67 (2019).
81. González, M. A. *et al.* A survey of tire-breeding mosquitoes (Diptera: Culicidae) in the Dominican Republic: considerations about a pressing

issue. *Biomédica* **40**, (2020).

82. *IX Censo Nacional De Población Y Vivienda*. vol. 1 (2012).
83. *PLISA Health Information Platform for the Americas*. https://www.paho.org/data/index.php/en/.
84. *Dominican Republic: Human Development Indicators*. http://hdr.undp.org/en/countries/profiles/DOM.
85. Kraemer, M. U. G. *et al.* The global compendium of Aedes aegypti and Ae. albopictus occurrence. *Sci Data* **2**, 150035 (2015).
86. Ferguson, N. M. *et al.* EPIDEMIOLOGY. Countering the Zika epidemic in Latin America. *Science* **353**, 353–354 (2016).
87. Lessler, J. *et al.* Times to key events in Zika virus infection and implications for blood donation: a systematic review. *Bull. World Health Organ.* **94**, 841–849 (2016).
88. Yasuno, M. & Tonn, R. J. A study of biting habits of Aedes aegypti in Bangkok, Thailand. *Bull. World Health Organ.* **43**, 319–325 (1970).
89. Trpis, M. & Hausermann, W. Dispersal and other population parameters of Aedes aegypti in an African village and their possible significance in epidemiology of vector-borne diseases. *Am. J. Trop. Med. Hyg.* **35**, 1263–1279 (1986).
90. Li, M. I., Wong, P. S. J., Ng, L. C. & Tan, C. H. Oral susceptibility of Singapore Aedes (Stegomyia) aegypti (Linnaeus) to Zika virus. *PLoS Negl. Trop. Dis.* **6**, e1792 (2012).
91. Wong, P.-S. J., Li, M.-Z. I., Chong, C.-S., Ng, L.-C. & Tan, C.-H. Aedes (Stegomyia) albopictus (Skuse): a potential vector of Zika virus in Singapore. *PLoS Negl. Trop. Dis.* **7**, e2348 (2013).
92. Trpis, M., Häusermann, W. & Craig, G. B. Estimates of Population Size, Dispersal, and Longevity of Domestic Aedes aegypti aegypti (Diptera: Culicidae) by Mark–Release–Recapture in the Village of Shauri Moyo in Eastern Kenya. *Journal of Medical Entomology* vol. 32 27–33 (1995).
93. Hugo, L. E. *et al.* Adult survivorship of the dengue mosquito Aedes aegypti varies seasonally in central Vietnam. *PLoS Negl. Trop. Dis.* **8**, e2669 (2014).
94. Aldstadt, J. *et al.* Space-time analysis of hospitalised dengue patients in rural Thailand reveals important temporal intervals in the pattern of dengue virus transmission. *Trop. Med. Int. Health* **17**, 1076–1085 (2012).
95. Majumder, M. S., Cohn, E., Fish, D. & Brownstein, J. S. Estimating a feasible serial interval range for Zika fever. *Bull. World Health Organ.* **10**, 1–6 (2016).
96. *Zika virus: Fact Sheet*. https://www.who.int/news-room/fact-sheets/detail/zika-virus (2018).
97. Riou, J., Poletto, C. & Boëlle, P.-Y. A comparative analysis of Chikungunya and Zika transmission. *Epidemics* **19**, 43–52 (2017).
98. Staples, J. E., Breiman, R. F. & Powers, A. M. Chikungunya fever: an epidemiological review of a re-emerging infectious disease. *Clin. Infect. Dis.* **49**, 942–948 (2009).
