# Supplementary material for: Asynchronicity of endemic and emerging mosquito-borne disease outbreaks in the Dominican Republic
Source: Nat Commun. 2021 Jan 8;12:151. doi: 10.1038/s41467-020-20391-x (PMC7794562; doi:10.1038/s41467-020-20391-x)
Supplement: Supplementary file 3 — Reporting Summary [file 41467_2020_20391_MOESM3_ESM.pdf]

nature research

Corresponding author(s): Mary Petrone

Last updated by author(s): Nov 24, 2020

Reporting Summary

Nature Research wishes to improve the reproducibility of the work that we publish. This form provides structure for consistency and transparency in reporting. For further information on Nature Research policies, see our Editorial Policies and the Editorial Policy Checklist.

Statistics

For all statistical analyses, confirm that the following items are present in the figure legend, table legend, main text, or Methods section.

n/a	Confirmed	
x		The exact sample size (n) for each experimental group/condition, given as a discrete number and unit of measurement
x		A statement on whether measurements were taken from distinct samples or whether the same sample was measured repeatedly
	x	The statistical test(s) used AND whether they are one- or two-sided<br>*Only common tests should be described solely by name; describe more complex techniques in the Methods section.*
	x	A description of all covariates tested
	x	A description of any assumptions or corrections, such as tests of normality and adjustment for multiple comparisons
x		A full description of the statistical parameters including central tendency (e.g. means) or other basic estimates (e.g. regression coefficient) AND variation (e.g. standard deviation) or associated estimates of uncertainty (e.g. confidence intervals)
	x	For null hypothesis testing, the test statistic (e.g. F, t, r) with confidence intervals, effect sizes, degrees of freedom and P value noted<br>*Give P values as exact values whenever suitable.*
x		For Bayesian analysis, information on the choice of priors and Markov chain Monte Carlo settings
x		For hierarchical and complex designs, identification of the appropriate level for tests and full reporting of outcomes
	x	Estimates of effect sizes (e.g. Cohen's d, Pearson's r), indicating how they were calculated

Our web collection on statistics for biologists contains articles on many of the points above.

Software and code

Policy information about availability of computer code

Data collection	No custom software was used to collect the data in our study.
Data analysis	Data were analyzed using Rv4.0.0., Prism v8.4.2, and MVSE (R package). R scripts are available at https://github.com/grubaughlab/Paper_arbovirus_Epi_DR.

For manuscripts utilizing custom algorithms or software that are central to the research but not yet described in published literature, software must be made available to editors and reviewers. We strongly encourage code deposition in a community repository (e.g. GitHub). See the Nature Research guidelines for submitting code & software for further information.

Data

Policy information about availability of data

All manuscripts must include a data availability statement. This statement should provide the following information, where applicable:
- Accession codes, unique identifiers, or web links for publicly available datasets
- A list of figures that have associated raw data
- A description of any restrictions on data availability

With the exception of global AaS and surveillance data, source data are available with this paper (https://github.com/grubaughlab/Paper_arbovirus_Epi_DR). Surveillance data are available upon solicitation of the Dominican Republic Ministry of Health SAIP (Solicitud de Acceso a Informacion Publica/Solicitation of Publicly Available Information; website: saip.gob.do). Global AaS values may be available upon request.

Field-specific reporting

Please select the one below that is the best fit for your research. If you are not sure, read the appropriate sections before making your selection.

☐ Life sciences ☐ Behavioural & social sciences ☒ Ecological, evolutionary & environmental sciences

For a reference copy of the document with all sections, see nature.com/documents/nr-reporting-summary-flat.pdf

Ecological, evolutionary & environmental sciences study design

All studies must disclose on these points even when the disclosure is negative.

Study description	This is a descriptive epidemiological study of three mosquito-borne viruses (dengue, chikungunya, and Zika) that caused a total of five outbreaks in the Dominican Republic between 2012 and 2018. We used descriptive statistics to compare the timing and demography of the cases reported during each outbreak. We then estimated the weekly transmission potential of Aedes aeygpti mosquitoes over this time period and compared the resulting curves to those of the reported cases. Next, we estimated the effective reproduction number for each of the outbreaks to the hypothesis that the two emerging virus outbreaks occurred earlier in the year than the three dengue outbreaks due to a larger susceptible population. We ruled out the possibility that local climate and land use drove the heterogeneous distribution of cases across the country using an Aedes aegypti suitability score. We used linear regression to demonstrate that certain provinces routinely experience a higher burden of dengue cases, but this was not predictive of the burden of emerging virus disease cases. Finally, we calculated the percent case fatality rate among cases reported during each season and found that this metric was higher for dengue cases reported during "inter-epidemic" periods.
Research sample	For this study, we used all of the reported cases of dengue, chikungunya, Zika, and febrile illness collected by the Ministry of Health between 2012 and 2018. Specifically, data for suspected and confirmed cases of dengue, chikungunya, and Zika reported between 2012-2018 were extracted from the Dominican Republic Ministry of Health Weekly Reports (digepi.gob.do). Depersonalized demographic and clinical characteristics of cases were solicited from the National Statistics Directorate of the Ministry of Health (MoH) (saip.gob.do). Data were organized by date of onset of symptom report. Province- and municipality-level weekly acute febrile illness data from 2012-2018 were solicited from the National Epidemiology Directorate of the Ministry of Health (saip.gob.do), which collects the data as part of a passive surveillance system. In 2014, the Ministry of Health increased its efforts to identify febrile illness cases<br><br>The median age of each group was as follows: 12.5 (dengue), 28 (chikungunya), and 30 (Zika). Approximately 44% of dengue cases, 61% of chikungunya cases, and and 74% of Zika cases were female. See Data collection for a description of the dataset and its source.
Sampling strategy	We used all the case data provided as described below with limited exclusions (see Data exclusions). Sampling was not required for our climatological analyses.
Data collection	Case data were collected by the Ministry of Health in weekly reports. Specifically, the National Epidemiology Directorate of the MOH collects dengue case reports by passive surveillance in a weekly manner in its Epi 1 form within their Sistema Nacional de Vigilancia Epidemiológica (SINAVE) Digital Platform for every healthcare setting across the country. These data were collected in weekly reports submitted to their website (digepi.gob.do) and organized by province and week of reported cases. The data collected by the MoH includes age, sex, province and municipality of residence, date of symptom onset, clinical outcome, and symptomatology. The MoH included mandatory reports from suspected and confirmed chikungunya infection through 2014 and Zika virus throughout 2016, without continuing to do so thereafter. Febrile illness case data were collected through passive surveillance by the MoH.<br><br>These data were de-identified and combined with demographic and clinical information provided through the National Statistics Directorate of the Ministry of Health. Dengue cases were collected through a passive reporting system. Zika and chikungunya were mandatory reportable diseases in 2016 and 2014, respectively. Province- and municipality-level weekly acute febrile illness data from 2012-2018 were solicited from the National Epidemiology Directorate of the Ministry of Health. Daily climatological variables for mean temperature and relative humidity for 5 cities (Santo Domingo, Distrito Nacional; Barahona, Barahona; La Union, Puerto Plata; Cabrera, María Trinidad Sánchez; Santiago, Santiago) was collected from the National Meteorology Office (ONAMET) database (saip.gob.do) from January 2012 through December 2018. Hourly climatological data for Bonao, Monseñor Nouel for the same timeframe were obtained from openweathermap.org.<br><br>Population data were extracted from the 9th National Population and Household Census. This census was conducted in 2010 by the National Statistics Office (National Statistics Office, 2012). Reported cases of dengue, chikungunya, and Zika for Haiti, Bolivia, Jamaica, and Venezuela were extracted from the Pan-American Health Organization website and PLISA database (PAHO, 2020d). Dengue and Zika cases, which were reported by epidemiological week, were aggregated by month to allow for a direct comparison to chikungunya cases, which were reported by month.
Timing and spatial scale	Dengue case data were collected between January 2012 and December 2018. Zika case data were collected between December 2015 and August 2016. Chikungunya case data were collected between January 2014 and December 2014. These data were collected daily on the municipality level for the entirety of the Dominican Republic by the Ministry of Health.
Data exclusions	For cases of dengue, Zika, and chikungunya we excluded individuals with a reported age over 100 years old because life expectancy in the Dominican Republic is 73 years old. We therefore assumed ages reported to be over 100 were most likely due to typographical errors. This exclusion was made before analyses were conducted.

Reproducibility	We repeated our analyses twice to ensure reproducibility. Replicate analyses did not produce qualitatively different results. All code and source data are available on GitHub.
Randomization	Randomization was not relevant to this study because we were not comparing outcomes among groups of organisms/animals/ humans.
Blinding	Blinding was not relevant to this study because we were not investigating the effect of an intervention. This was an observational study, and it was therefore necessary that we clearly identified individuals who had been diagnosed with either dengue, Zika, chikungunya, or febrile illness as we planned our analysis.

Did the study involve field work? ☐ Yes ☒ No

Reporting for specific materials, systems and methods

We require information from authors about some types of materials, experimental systems and methods used in many studies. Here, indicate whether each material, system or method listed is relevant to your study. If you are not sure if a list item applies to your research, read the appropriate section before selecting a response.

Materials & experimental systems

n/a	Involved in the study
☒	☐ Antibodies
☒	☐ Eukaryotic cell lines
☒	☐ Palaeontology and archaeology
☒	☐ Animals and other organisms
☒	☐ Human research participants
☒	☐ Clinical data
☒	☐ Dual use research of concern

Methods

n/a	Involved in the study
☒	☐ ChIP-seq
☒	☐ Flow cytometry
☒	☐ MRI-based neuroimaging
